# Scaffold Modifications in Erythromycin Macrolide Antibiotics. A Chemical Minireview

**DOI:** 10.3390/molecules25173941

**Published:** 2020-08-28

**Authors:** Kjell Undheim

**Affiliations:** Department of Chemistry, University of Oslo, 0315 Oslo, Norway; kjell.undheim@kjemi.uio.no

**Keywords:** semisynthesis, total synthesis, ketolides, medicinal chemistry, macrolide scaffold, clarithromycin, antibacterials

## Abstract

Clarithromycin and congeners are important antibacterial members of the erythromycin A 14-membered macrocyclic lactone family. The macrolide scaffold consists of a multifunctional core that carries both chemically reactive and non-reactive substituents and sites. Two main approaches are used in the preparation of the macrolides. In semisynthesis, the naturally occurring macrocycle serves as a substrate for structural modifications of peripheral substituents. This review is focused on substituents in non-activated positions. In the total synthesis approach, the macrolide antibiotics are constructed by a convergent assembly of building blocks from presynthesized substrates or substrates prepared by biogenetic engineering. The assembled block structures are linear chains that are cyclized by macrolactonization or by metal-promoted cross-coupling reactions to afford the 14-membered macrolactone. Pendant glycoside residues are introduced by stereoselective glycosylation with a donor complex. When available, a short summary of antibacterial MIC data is included in the presentations of the structural modifications discussed.

## 1. Introduction

**Figure 1:**
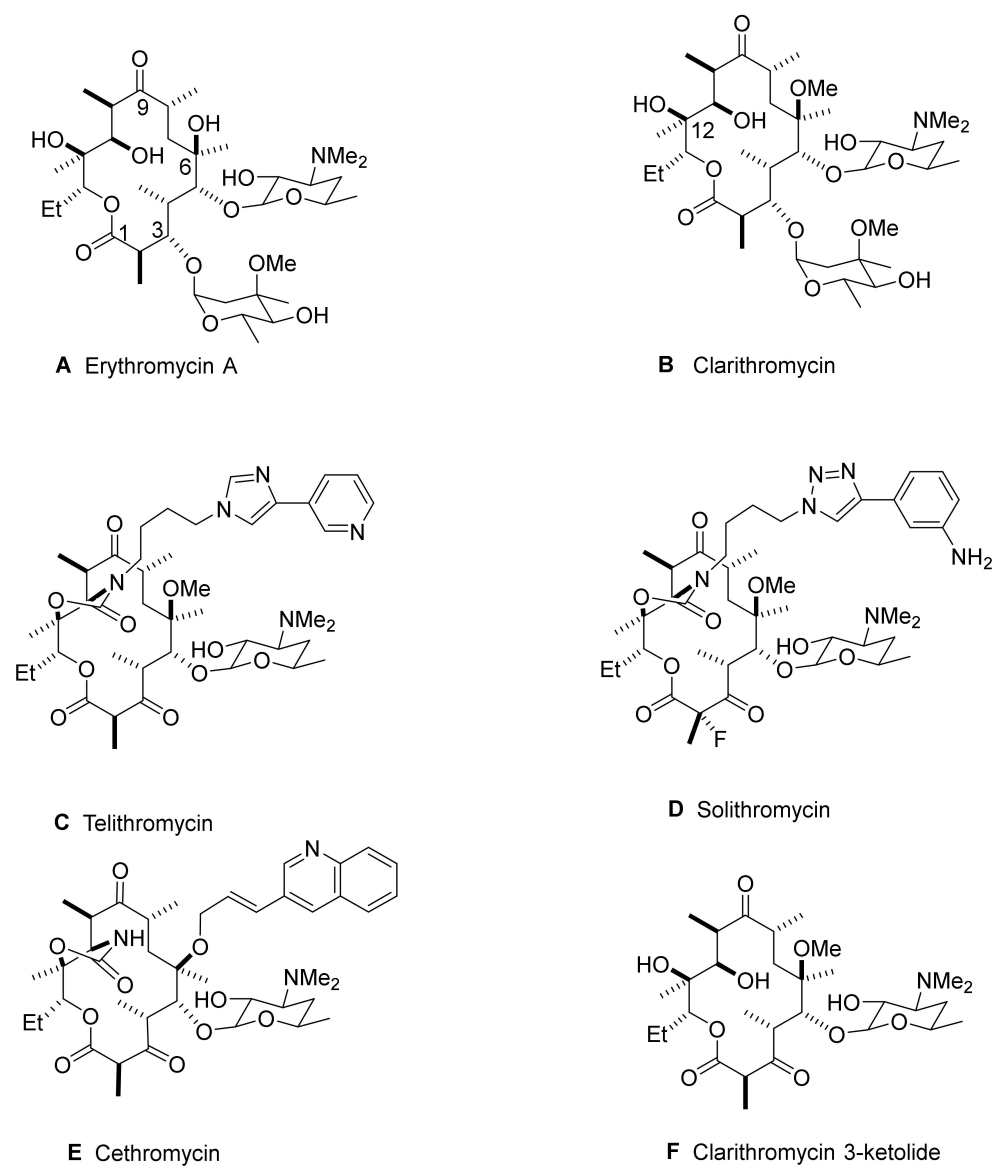
Erythromycin A (**A**), Clarithromycin (**B**), Telithromycin (**C**), Solithromycin (**D**), Cethromycin (**E**), and Clarithromycin 3-ketolide (**F**).

Clarithromycin (B, Figure 1) and congeners are antibacterials from the erythromycin A 14-membered lactone family. The macrolides exert their drug action by inhibition of bacterial protein synthesis in sensitive pathogens [1,2,3]. Members of each antibiotic class share a common core structure or scaffold. Several reports describe efforts to improve antimicrobial efficacies, widen the microbial spectra, and improve activity against pathogens that have become partly resistant to the macrolides. In most structural investigations, the core of the antibiotic is left intact to preserve the natural activity of the scaffold. The chemical groups at the periphery of the scaffold are modified. The multifunctional erythromycin scaffold carries chemically reactive as well as chemically inert substituents. This review describes work on modifications of chemically inert carbon substituents and non-activated sites in the scaffold congeners of clarithromycin ketolides [4,5,6].

**Scheme 1:**
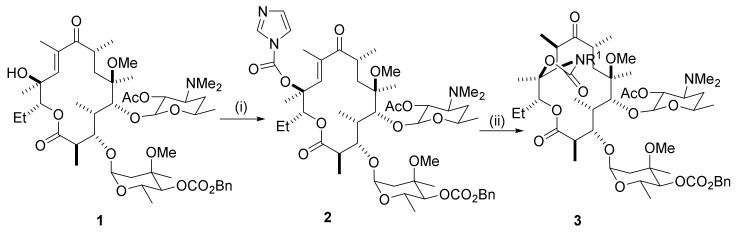
Reaction and conditions: (**i**) CDI, NaH, DMF, THF, 0 °C to rt, 4–5 h; (**ii**) R^1^NH_2_, DMF, or MeCN (10% aq), rt, 1–3 d. Abbreviation: CDCI, 1,1′-carbonyldiimidazole.

The semisynthetic drug, clarithromycin, is a 6-methyl ether of the parent 14-membered erythromycin A. Removal of the 3-(*L*)-desosamine sugar residue in erythromycin and oxidation of the resultant free 3-hydroxy group afford highly active 3-oxo antibacterials. The potencies of some of these compounds are further improved after annulation reactions that afford cyclic 11,12-carbamates, where the 11,12-cyclic carbamate linkage occupies the positions of the two 11,12-hydroxy groups in erythromycin (Scheme 1). The methodology for the preparation of cyclic 11,12-carbamate macrolides was developed by Baker and coworkers [7].

Erythromycin derivatives are preferably prepared by semisynthetic methodology. Total synthesis has remained a challenge. Recent work, however, has opened up for total syntheses of modified structures (vide infra). Intermediates from biogenetic engineering are incorporated into the products.

The presentation in this report is arranged according to the increase in numbering of the sites in the macrolide core, starting with the ester carbonyl carbon as the 1-position (Structure **A** in Figure 1). Clarithromycin is formally formed by replacement of the 6-hydroxy group in erythromycin by a methoxy group. In telithromycin (**C**) and solithromycin (**D**), the 3-glycocyl function has been cleaved by hydrolysis and the resultant 3-hydroxy group oxidized to afford the 3-ketolide. In a similar way, cethromycin (**E**) is a 3-ketolide, but the 6-hydroxy group has been converted into an ether function, where the *O*-sidechain is bridged by an allyl group onto the 3-position in quinoline. Clarithromycin 3-ketolide (**F**) is available as above from clarithromycin.

## 2. Synthesis

A series of reactions, starting at the C12-hydroxy group in a substrate such as compound **1** (Scheme 1) to afford a cyclic C11,C12-carbamate **3**, is frequently referred to as the Baker protocol. The carbamoyl reaction of the 10,11-anhydroerythromycin substrate **1** (Scheme 1) is carbamoylated at the 12-hydroxy group by carbonyl diimidazole (CDI) using NaH in DMF or THF to afford the carbamate **2**. A subsequent treatment with aqueous ammonia in acetonitrile leads to a 2-step cyclization reaction and formation of 11-deoxy-11-carboxyamino-6-*O*-methylerythromycin A 11,12-cyclic ester **3**. The Michael reaction requires base catalysis and the rate of addition is fastest in polar solvents such as 10% aqueous acetonitrile or DMF. With primary amine reactants, a variety of *N*-substituted products (**3**) is formed. The major product from the intramolecular Michael reaction has the natural (*R*)-configuration of erythromycin at the C10-position. The stereochemistry at C10 can be established by NMR spectroscopy (vide infra) [7].

### 2.1. C2-Derivatives

**Scheme 2:**
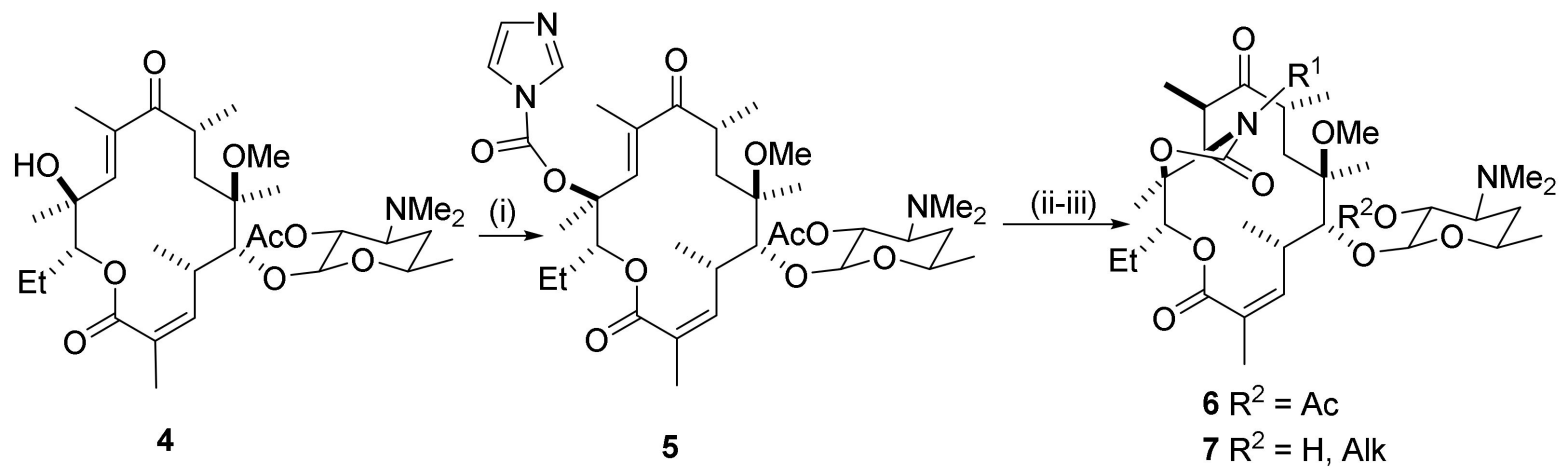
Reagents and conditions: (**i**) CDI, NaH, DMF, THF, 0 °C to rt, 4–5 h; (**ii**) R^1^NH_2_, DMF, or MeCN (10% aq.), rt, 1–3 d; (**iii**) MeOH, rt, 14 h.

The acylimidazolide **5** is prepared in a similar manner from the corresponding C12-hydroxy 2,3- and 10,11-diene **4** by reactions with CDI (Scheme 2) [8]. Further reactions with ammonia or primary amines in aqueous acetonitrile or DMF afford cyclocondensation and formation of the fused cyclocarbamate **6**. Methanolysis at room temperature cleaves off the 2′-protecting group. The product **7** is an epimeric mixture at C10-position, but frequently, the product formation shows high preference for the natural (10*R*)-epimer. Epimeric mixtures can be isomerized under basic conditions towards the more stable (10*R*)-isomer. *N*-Alkylated carbamates are obtained when a primary amine is used instead of ammonia. Reductive alkylation of the carbamate nitrogen after an ammonia cyclization will also yield *N*-substituted derivatives.

Antibacterials: The cyclic carbamates show good antibacterial activity against inducibly MLS-resistant *S. aureus A 5177*, against macrolide-resistant *S. Pyogenes PIU* 2548 and *S*. *pneumonia* 5649 m, but are inactive against Gram-negative organisms. The C10-*epi* analogues are significantly less active than their natural C10-counterparts. In vivo activity is inferior to the activity of clarithromycin.

**Scheme 3:**
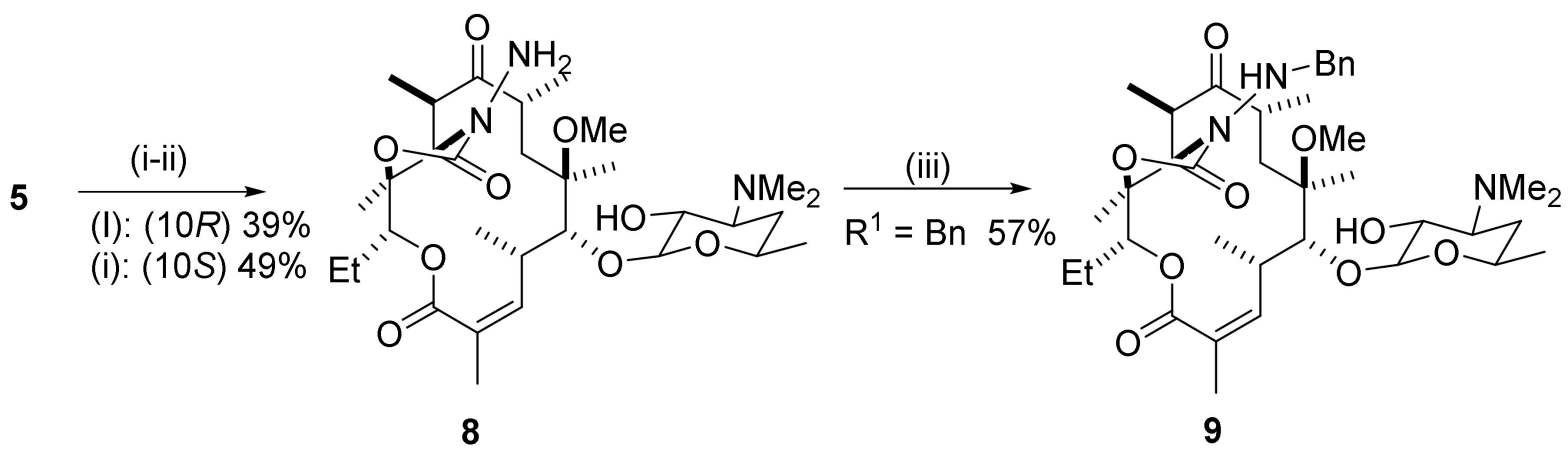
Reagents and conditions: (**i**) NH_2_NH_2_, DMF, 60 °C; (**ii**) MeOH, rt; (**iii**) PhCHO, NaBH_3_CN, AcOH, MeOH, rt, 3 h.

Carbazates are formed when hydrazine is the amine reactant. The carbamate **5** reacts with hydrazine in DMF to afford 2,3-anhydro-5-*O*-desosaminyl-11-hydrazo-6-*O*-methylerythronalide A 11,12-carbamate **8** (Scheme 3). Formation of an epimeric mixture of the (10*R*)-carbazate (39%) and the (10*S*)-carbazate (49%) **8** is shown [9]. Reductive alkylations with aldehydes using NaBH_3_CN and acetic acid afford *N*-alkylated products. With benzaldehyde as the reactant, the *N*-benzyl-2,3-anhydro derivative **9** is formed from the carbazate **8** (Scheme 3) [10].

Antibacterials: The antibacterial potency of 11,12-cyclic carbazates is comparable to the potency of erythromycin A. *N*-Alkylation improves their potency as antibacterials. C10-epimers are generally less active than their natural C10-epimers. The compounds are moderately active in in vivo studies in mice [9,10].

### 2.2. Reactivity of the C2-Methyl Group

**Scheme 4:**
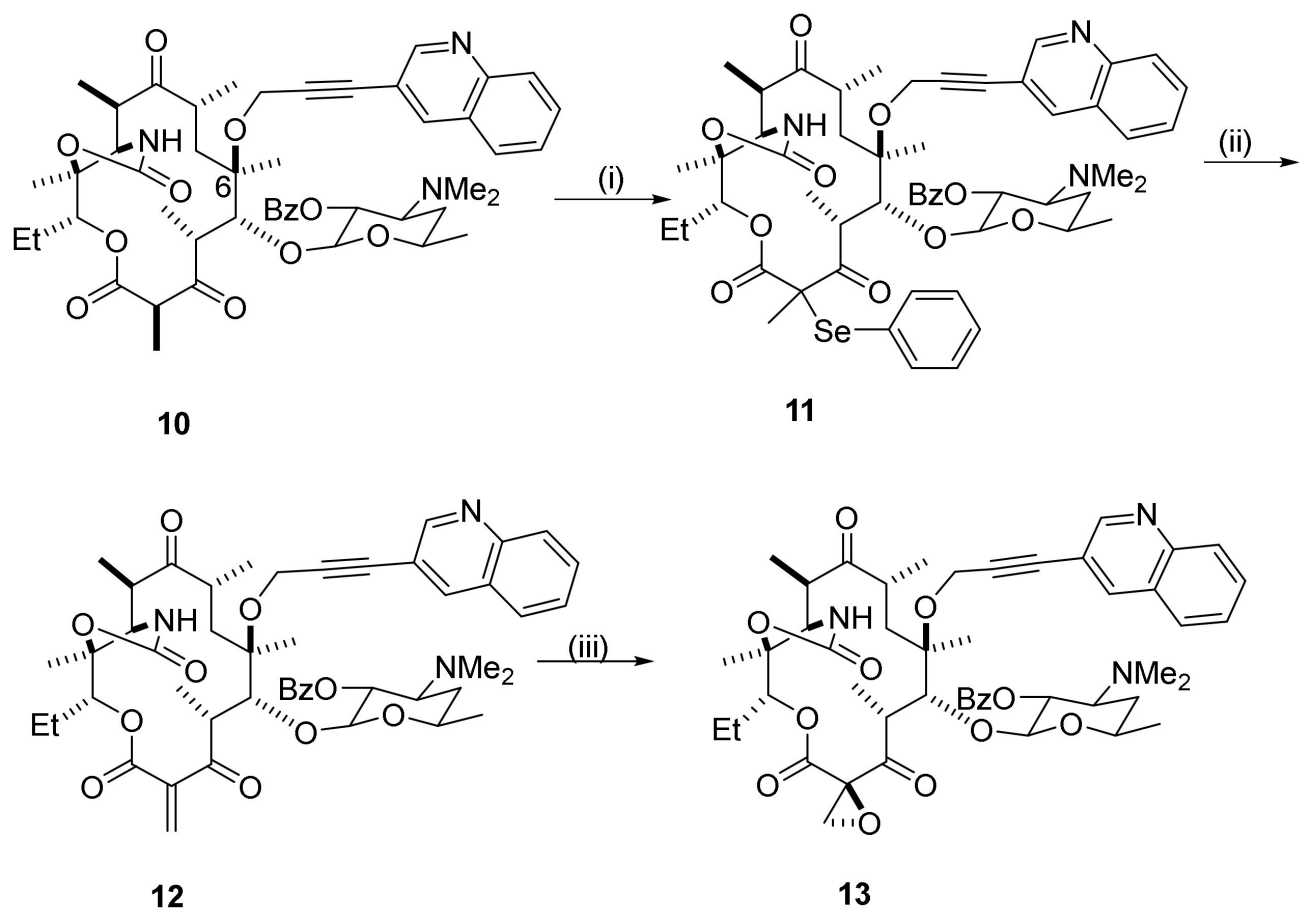
Reagents and conditions; (**i**) NaH, DMF, PhSeCl, 0 °C, 3 h; (**ii**) Oxone, THF, H_2_O, rt, 26 h; (**iii**) H_2_O_2_. Abbreviation: Oxone, potassium peroxymonosulfate.

The C2-position in the 1,3-dioxo substrate **10** is chemically activated by the adjacent oxo groups and is readily enolized. The 6-*O*-protected ketolide **10**, as an enolate, reacts readily with electrophilic reagents to afford 2-halo, 2-benzenesulfenyl, or 2-benzeneselenyl intermediates (**11**) (Scheme 4). The latter are substrates for nucleophilic displacements [11]. Sulfides, and especially selenides, are highly activated by oxidation, and the oxides readily suffer base promoted elimination with formation of a 2-methylene product **12** (Scheme 4). Further oxidations of the C2-methylene compound **12** using hydrogen peroxide reagents afford diols as well as epoxide **13**.

Antibacterials: The compounds are, in general, active antibacterials. MIC values are available.

### 2.3. Fluoro Derivatives

**Scheme 5:**
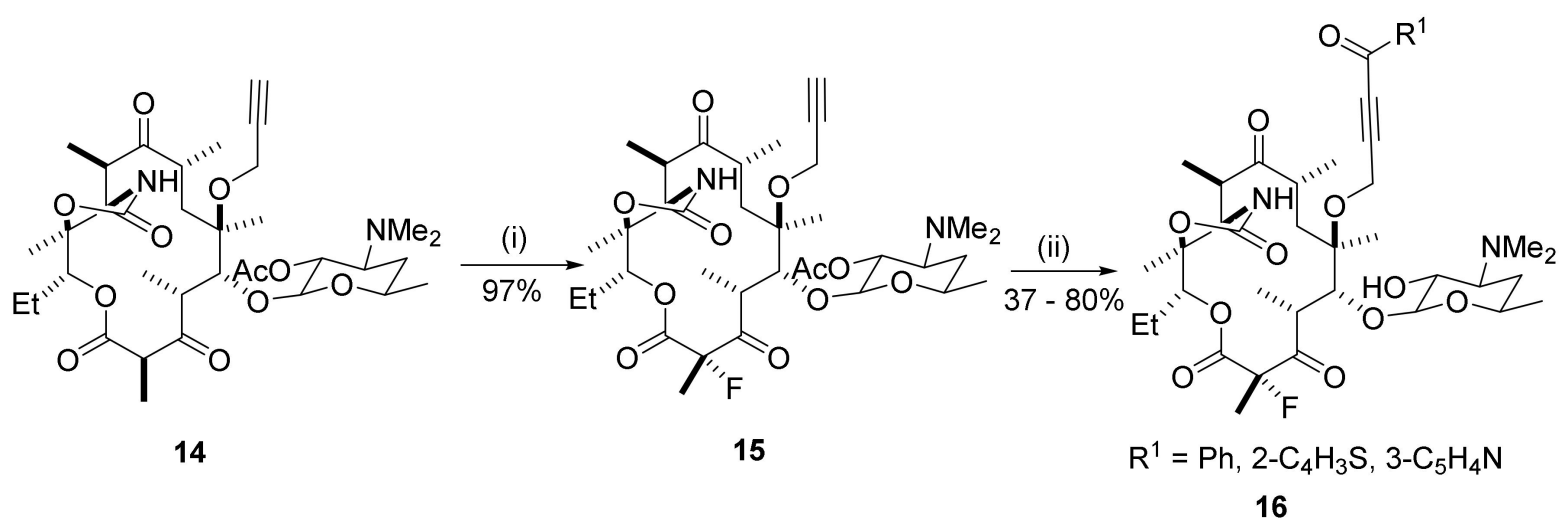
Reagents and conditions; (**i**) (a) NaH, DMF, 0 °C, 1 h, (b) (PhSO_2_)_2_NF, −5 °C, 3 h; (**ii**) R^1^COBr(Cl), PPh_3_, CuI, NEt_3_, MeCN, 80 °C; (**iii**) MeOH, rt.

In medicinal chemistry, fluorine as a hydrogen bioisostere is used to alter the polarity, lipophilicity, or metabolic stability of a drug. The inclusion of fluorine may be beneficial for activity, protein binding, and cell penetration. Several drugs contain fluoro substituents. Fluorides are included in this review. In a macrolactone 3-ketolide, the C2-position is activated for electrophilic substitutions after initial enol ether or ester formation. The enolized substrate is halogenated and fluorinated. The 2-fluoro derivative is a stable compound, whereas corresponding chloro or bromo halides readily undergo substitution or elimination reactions. The C2-F substituent prevents the 3-keto function from enolization. A synthesis of a 2-fluoro-6-*O*-propargyl-11,12-cabamate **16** is shown in Scheme 5 [12]. The 6-*O*-propargylic ketones and electron-deficient 6-propargylic aromatic derivatives resist enolization of the β-keto ester. The propargyl 2-fluoroketolide **15** in Scheme 5 is prepared directly from enol species arising from the ketone **14** by treatment with NaH under ionic conditions using *N*-fluorobenzene sulfonamide for the fluorination. The product **15** is a single diastereomer. Trans-coupling reactions with acyl bromides or chlorides are promoted by Pd-catalysis under Sonogashira conditions to deliver carbocyclic or heterocyclic alkynyl ketones. Subsequent deacylation of the 2′-protected species is effected by dissolution of the product in cold methanol to afford the target compound **16**.

Antibacterials: The MIC data for the compounds are consistent with high antibacterial activity.

**Scheme 6:**
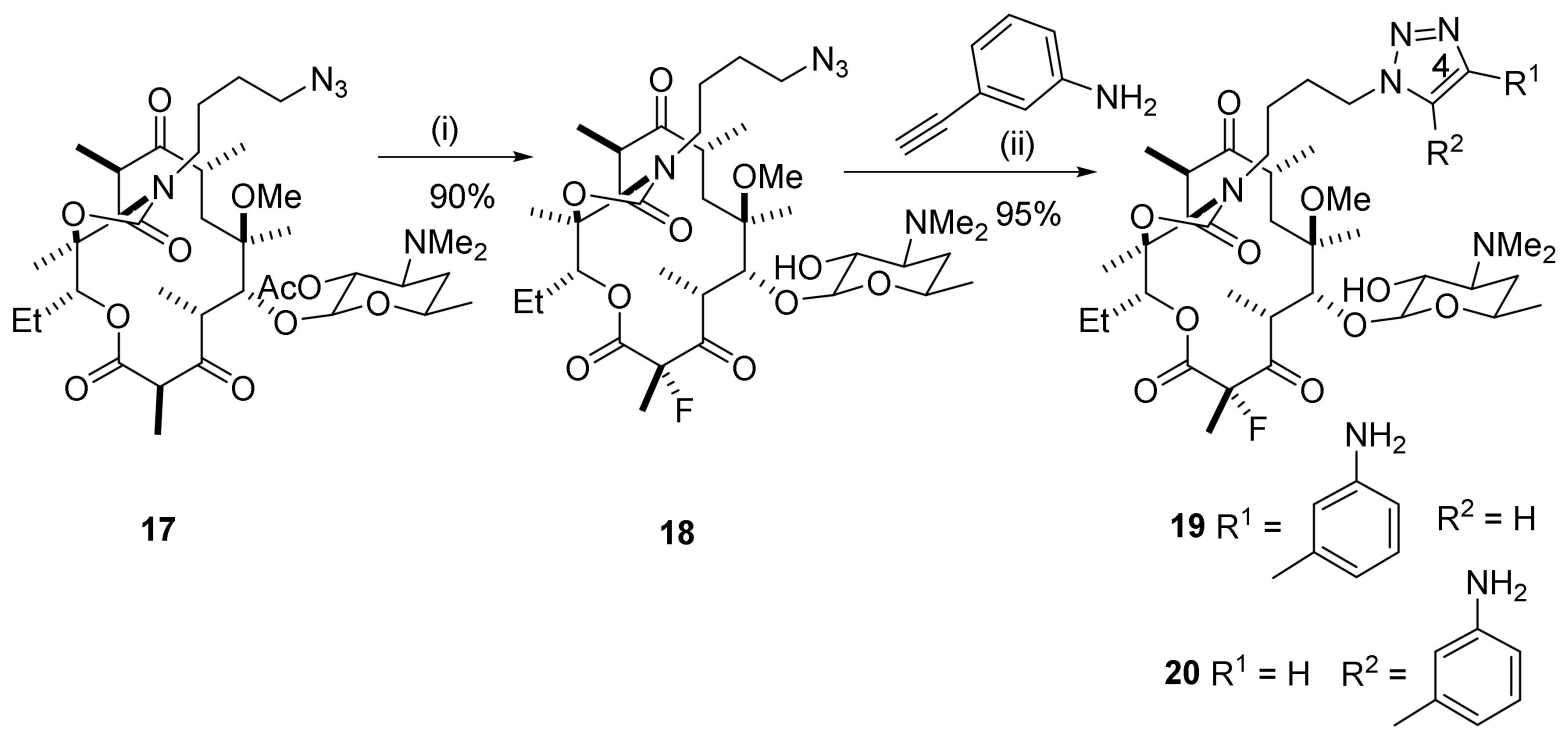
Reagents and conditions. (**i**) NaH, (PhSO_2_)_2_NF, THF; (**ii**) MeOH, rt; (**iii**) CuI (cat), toluene, 86 °C.

Solithromycin (**D**, Figure 1) is a powerful 2-fluoroantibiotic that can be prepared by click chemistry from the azide **17** and 3-ethynylaniline. The (3 + 2) dipolar cycloaddition is effected by Cu(I)-catalysis (Scheme 6) [13,14]. The catalyst is generated in situ from CuSO_4_, sodium ascorbate, and the terminal alkyne in *t*BuOH:water (1:1) at rt, 24 h, yield 70–90% of the anti-(1,4)-triazole **19**. 2-Fluoro-11-pendent-ω-azidobutyl-ketolides such as the azide **17** is converted to 4-substituted [1,2,3]-triazole as a single regional isomer by reaction with the corresponding acetylene to afford 2-fluoro-ketolide **19 [13]**. Hetaryl derivatives such as 3-thienyl derivatives are prepared in the same manner.

Antibacterials: Some of the compounds possess equipotent MIC values with solithromycin and are active against wild-type and resistant isolates of Gram-positive *S. aureus* strain, *E. coli* strains, and drug multiresistant pathogens.

Ribosome-templated azide-alkyne cycloadditions are achieved in situ by click chemistry in a similar manner. With ruthenium catalysis, the regiochemistry is changed from structure **19** to the syn-(1,5)-triazole **20** [14].

Antibacterials: Solithromycin is a very potent antibiotic. Four analogues from the ribosome-templated derivatives in situ display similar therapeutics indices as solithromycin.

### 2.4. 9-Aza Derivatives

**Scheme 7:**
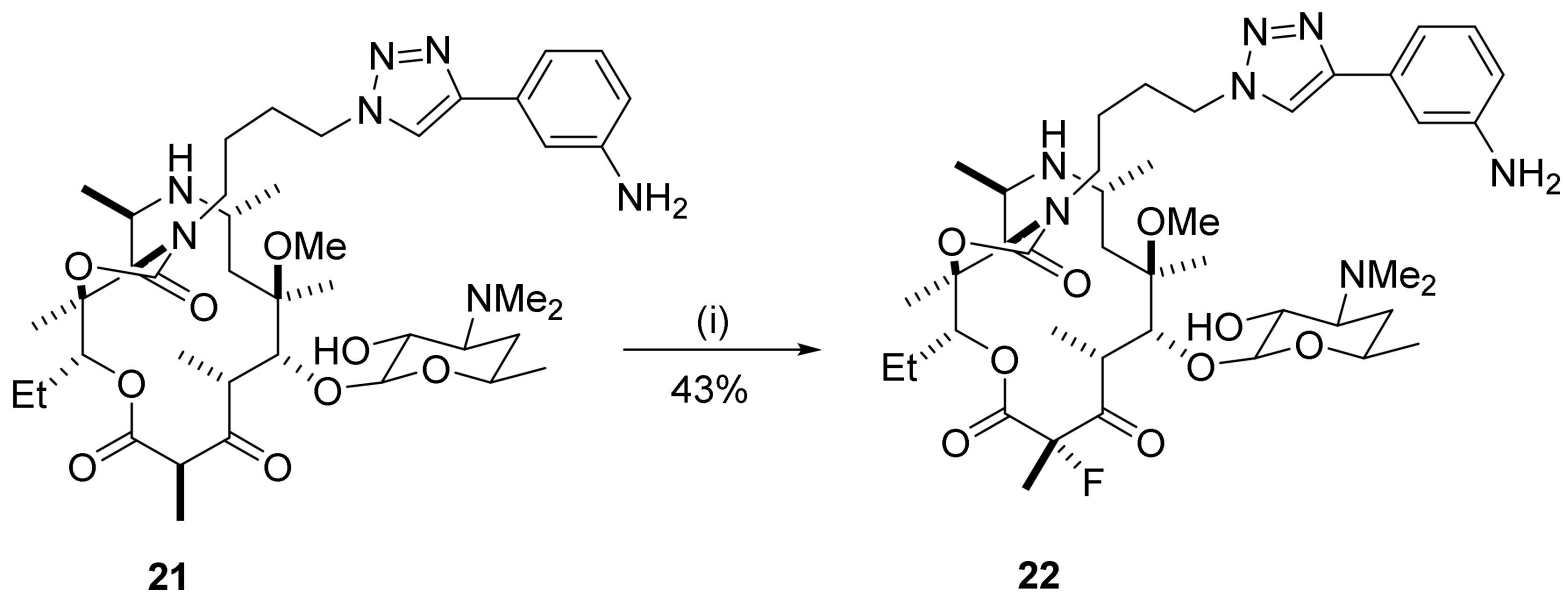
Reagents and conditions: (**i**) (PhSO_2_)_2_NF, *t*BuOK, −78 °C.

Replacement of the 9-oxo group in the macrolide skeleton by an amino nitrogen atom has no apparent influence on fluorination in the 2-position and fluorination of the 9-aza substrate **21** affords the 2C-fluoro product **22** (Scheme 7) [15].

### 2.5. Synthesis of 4-Desmethyl

**Scheme 8:**
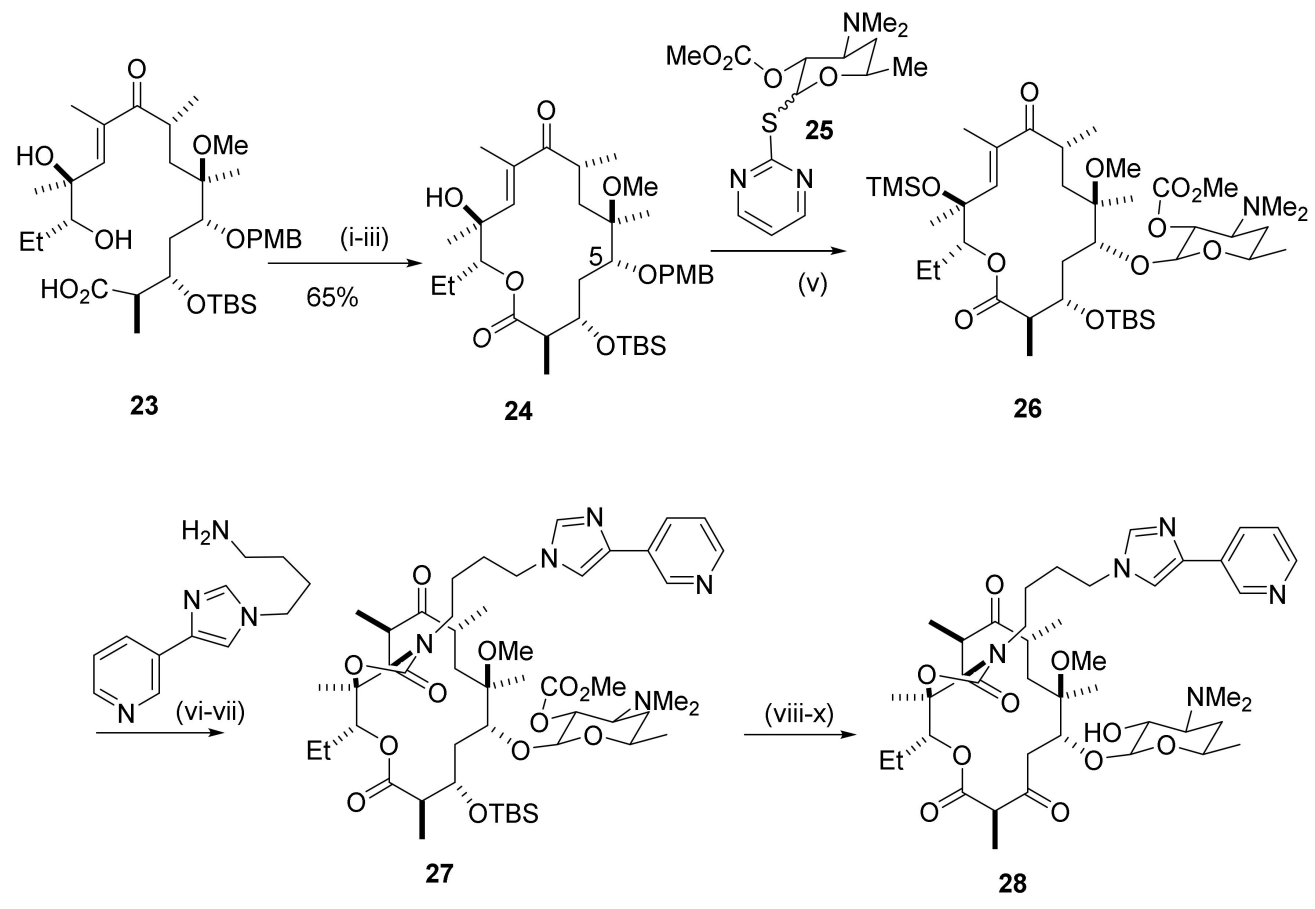
Reagents and conditions: (**i**) CCl_3_COCl, *i*PrNEt_2_, DMAP, benzene, 12 h; (**ii**) CeCl_3_.7H_2_O, NaBH_4_, MeOH, −15 °C to rt, 45 min; (**iii**) 2,6-lutidine, TMSOTf, CH_2_Cl_2_, −7 °C, 30 min: (**iv**) DDQ, H_2_O, CH_2_Cl_2_, 0 °C, 30 min; (**v**) AgOTf, DTBMT, 4 Å mol. sieve, toluene, CH_2_Cl_2_, 12 h; (**vi**) THF, pyridine, 0 °C to 15 °C, 3 h; (**vii**) DMP, CH_2_Cl_2_, 3 h; (**viii**) NaH, CDI, DMF, THF, −20 °C to 0 °C, 45 min; (**ix**) MeCN, H_2_O, 72 h¸ (**x**) TASF, DMF, H_2_O, 14 h; (**xi**) Me_2_S, NCS, NEt_3_, CH_2_Cl_2_, −20 °C; (**xii**) MeOH, rt, 14 h. Abbreviation: DTBMP, 2,6-di-tbutyl-4-methylpyridine; TASF, tris(dimethylamino)sulfonium difluorotrimethylsilicate; DMP, Dess-Martin periodinane.

Extensive synthetic organic chemistry is involved in the total syntheses of 14-membered macrolide scaffolds and falls outside the scope of this review. The presentation herein is restricted to syntheses that start at the step for the cyclization reactions and afford 14-membered macrolide scaffolds. The antibacterial importance of methyl groups hinged to the macrocycle in non-activated positions is determined indirectly after total syntheses of desmethyl macrolide scaffolds. Scheme 8 shows the preparation of the 4-desmethyl macrolide **28**. The telithromycin intermediate **24** is prepared from the linear hydroxy acid **23** by a macrolactonization reaction to afford **24** in 65% yield [16]. The hydroxy acid **23** is prepared by a multistep process. Stereoselective glycosylation of **24** with the donor of the desosamine sugar **25** affords the glycoside **26**. The Baker protocol subsequently delivers the macrolactone framework that affords the ketolide **28** [16].

Antibacterials: 4-Desmethyl telithromycin is equipotent with telithromycin against wild-type bacteria but is 4-fold less potent against the A2058G mutant.

### 2.6. 4,10-Didesmethyl-Telithromycin Derivatives

**Scheme 9:**
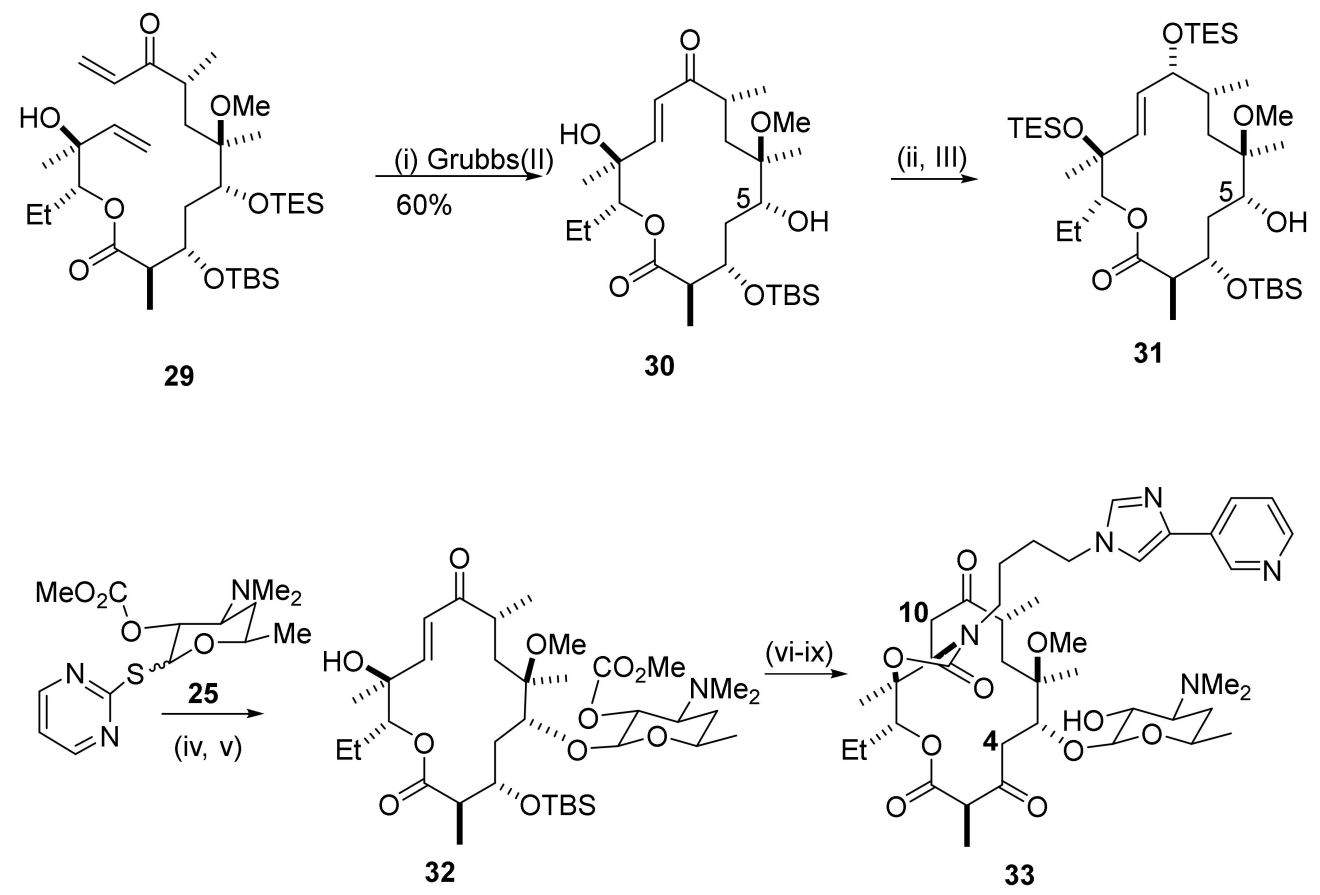
Reagents and conditions: (**i**) 20 Mol% Grubbs(II) catalyst; (**ii**) NaBH_4_, CeCl_3_.7H_2_O; (**iii**) TESCl, imidazole, (**iv**) AgOTf, DTBMP; (**v**) DMP, CH_2_Cl_2_: (**vi**) NaH, CDI, THF, DMF; (**vii**) amine; (**vii**) TAS, DMF, H_2_O; (**viii**) NCS, Me_2_S, NEt_3_; (**ix**) MeOH.

Total synthesis involves several reaction steps to afford a linear chemo and stereoselectively substituted substrate **29** for the synthesis of macrolide **33** without methyl groups in the 4- and 10-positions (Scheme 9). The intermediate **29** is cyclized by a Grubbs(II)-catalyzed RCM (ring closing metathesis) reaction to afford the cyclic structure **30** in 60% yield [17]. Reduction of the 9-oxo group and protection of the resultant hydroxy group afford intermediate **31** that is glycosylated using the desosamine donor **25** to generate intermediate **32.** The cyclic C11-C12 carbamate moiety is installed by reactions with CDI and NaH, and subsequently, by reactions with a 1,4 diaminobutane. Methanolysis removes the protecting groups in the product and delivers the desmethyl macrocycle **33**.

Antibacterials: 4,10-Didesmethyl telithromycin is less potent than telithromycin but four times more active than the 4,8,10-tridesmethyl congener.

### 2.7. 4,8,10-Tridesmethyl-Telithromycin Derivatives

**Scheme 10:**
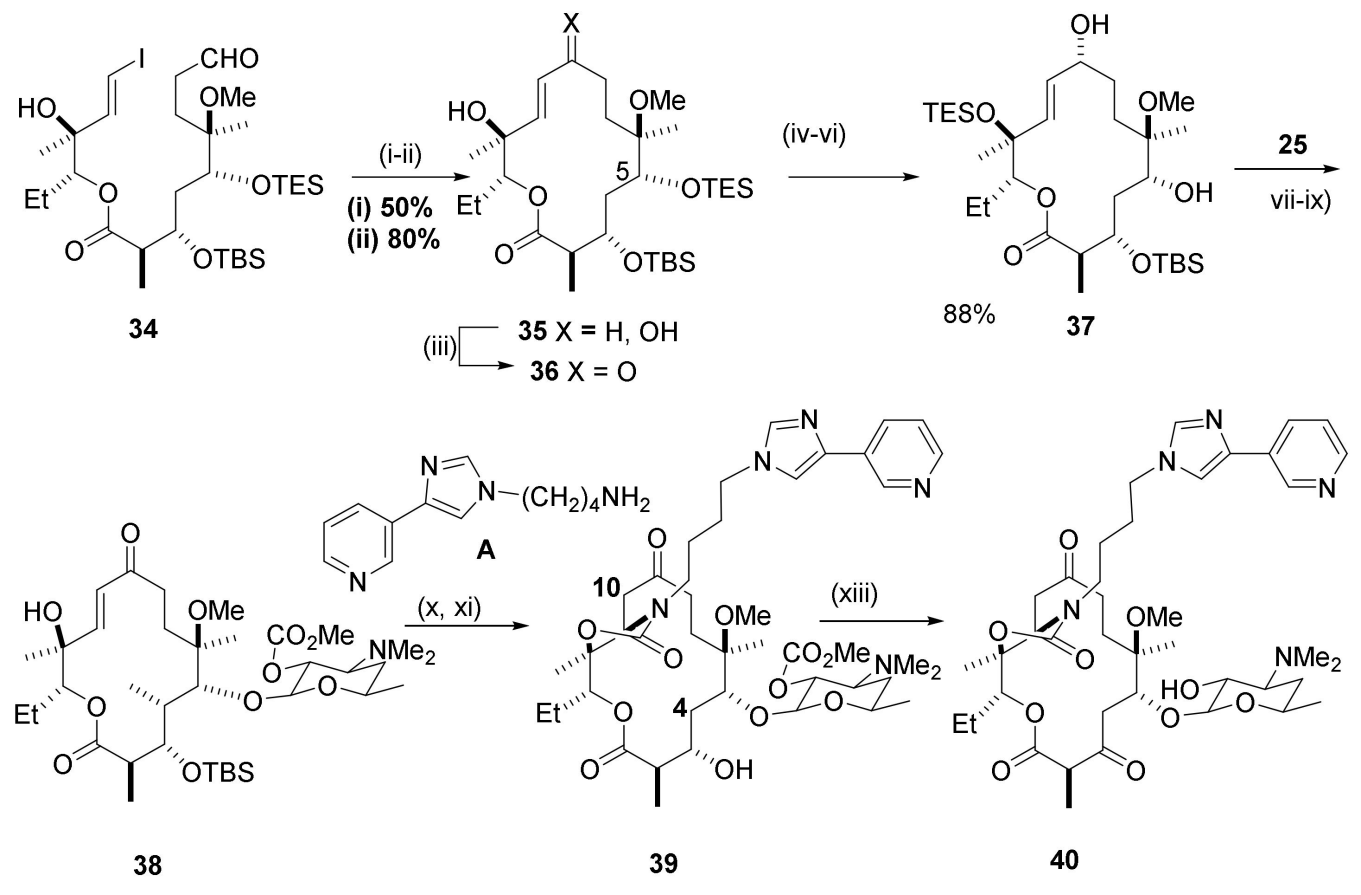
Reagents and conditions: (**i**) CrCl_2_, NiCl_2_(cat), DMSO; (**ii**) DMP; (**iii**) NaBH_4_, CeCl_3_.7H_2_O; (**iv**) TESOTf, 2,6-lutidine; (**v**) TsOH; (**vi**) TESCl, imidazole; (**vii**) **25**, AgOTf, DTBMP; (**viii**) 1 M TBAF, THF; (**ix**) DMP, CH_2_Cl_2_; (**x**) NaH, CDI, THF, DMF, then diamine (**A**); (**xi**) TASF, DMF, H_2_O; (**xii**) NCS, DMS, NEt_3_; (**xiii**) MeOH. Abbreviation: TES, triethylsilyl.

Total synthesis of (-)-4,8,10-tridesmethyl-telithromycin analogue of the telithromycin ketolide involves a ring closing reaction for construction of the 14-membered ring. Glycosylation by the donor complex **25**, the Baker sequential one-pot carbamoylation, and intramolecular amine reactions afford the analogue **40** (Scheme 10) [18].

Antibacterials: The products demonstrate antibacterial activity against several wild-types and resistant bacterial strains but are, in general, less potent than the parent telithromycin.

### 2.8. Cethromycin. Ketolides

**Scheme 11:**
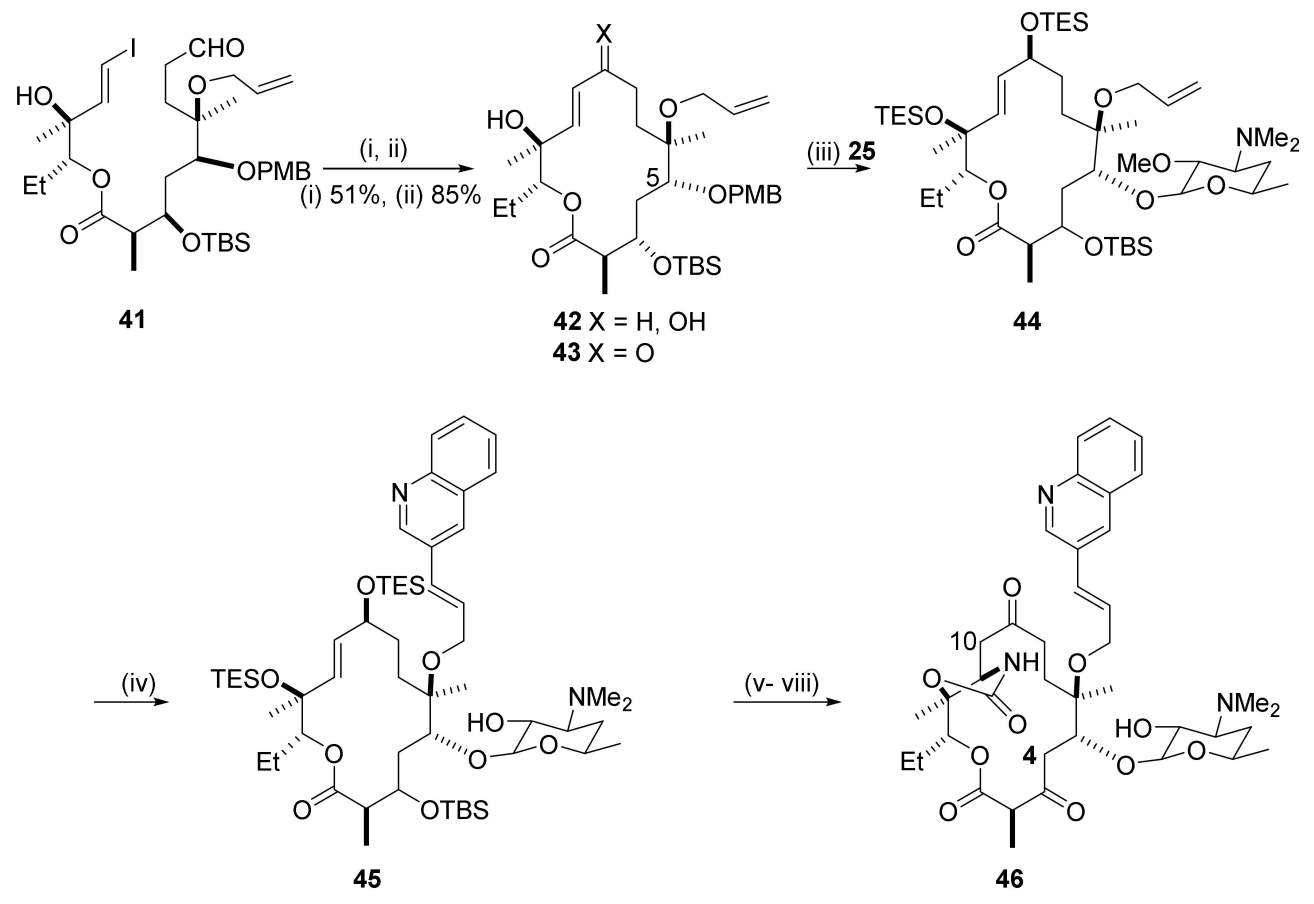
Reagents and conditions: (**i**) CrCl_2_, NiCl_2_ (100:1), DMSO; (**ii**) DMP, NaHCO_3_, CH_2_Cl_2_; (**iii**) AgOTf, DTBMP, 4 Å mol sieve, toluene, CH_2_Cl_2_, rt, 24 h; (**iv**) Pd(OAc)_2_, NaHCO_3_, 3-Br-quinoline, Bu_4_NCl, DMF, 80 °C; (**v**) TBAF, THF, 0 ^o^C, 15 min; (**vi**) DMP, CH_2_Cl_2_; (**vii**) NaH, CDI, DMF, THF, −20 °C–0 °C, (**viii**) NH_4_OH, MeCN, H_2_O.

The 14-membered macrolactone 4,8,10-tridesmethyl of cethromycin (**E,** Figure 1) is prepared from the linear chain substrate **41** that is synthesized by a multistep process (Scheme 11) [19]. Cyclization is achieved by the addition of CrCl_2_ and catalytic amount of NiCl_2_ to a solution of **41** in degassed DMSO with stirring at rt for 20 h. The product **42** (51%) is a 1:1 mixture of diastereomeric allylic alcohols at C9. Subsequent oxidation of **42** with DMP affords the 9-oxo product **43** that is glycosylated by a reaction with the thiopyrimidine desosamine donor **25** to afford **44**. In the installation of the cethromycin 3-quinolinyl sidechain, **44** is subjected to a modification of the Heck reaction with 3-bromoquinoline to afford **45** in 60% yield. Fluoride mediated cleavage of silyl ether protecting groups using TBAF and a subsequent DMP oxidation of the resulting hydroxy intermediate afford the corresponding 3,9-dioxo compound. The Baker protocol with NaH and CDI followed by ammonium hydroxide treatment afford the oxazolidinone **46**.

Antibacterials: The inhibitory activity against *E. coli* and *S. aureus* in the telithromycin series increases with the number of methyl groups. The desmethyl cethromycin analogue exhibits similar potency to that of telithromycin and is more active than the desmethyl telithromycin analogues against wild-type *E. coli* and A2058G mutant strains.

A summary of the synthetic achievements in the total synthesis program of desmethyl macrolide antibiotics is available [20].

Antibacterials: Activity data for the influence of the C4-, C8-, and C10-methyl groups in the macrolactones are available from screening programs against *E. coli* and *S. aureus* bacteria. All compounds tested are inactive against *E. coli.* A significant increase in antibacterial potency results from methyl group additions to the macrolactone scaffold (vide supra). This finding is attributed to changes in the macrolactone conformation caused by introduction of the C8- and C10-methyl groups that serve to rigidify the molecule [20].

### 2.9. Synthesis of C8-Fluoro Derivatives

**Scheme 12:**
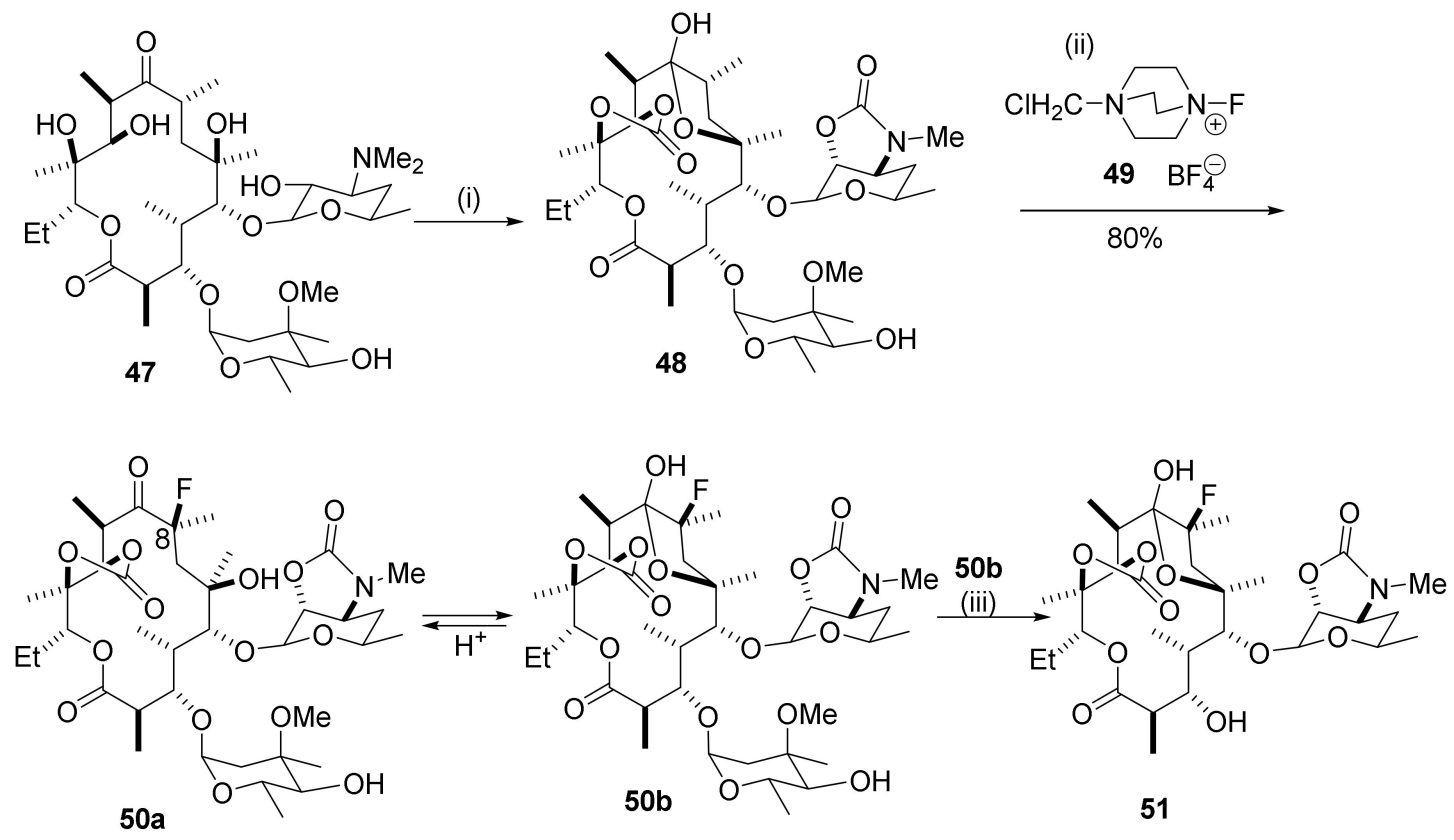
Reagents and conditions: (**i**) COCl_2_, pyridine, CH_2_Cl_2_, rt, 6 h; (**ii**) MeCN, H_2_O, rt, 40 min; (**iii**) AcOH, H_2_O, reflux, 30 min.

Fluorination in the 8-position in erythromycin is achieved via the cyclic carbamate **48**. The latter is prepared in a reaction between **47** and phosgene with pyridine as the base. In the acylation process, the sugar dimethylamino substituent loses one of the methyl groups (Scheme 12). 1-(Chloromethyl)-4-fluoro-1,4-diazabicyclo-[2,2,2]octane bistetrafluoroborate **49** (Selectfluor^TM^) is an effective agent for fluorination. In reactions with 8,9-anhydro-*N*-desmethylerythromycin A 2′,3′-carbamate-11,12-carbonate-6,9-hemiacetal, a mixture of the tautomers of hemiketal **50a** and the hydroxy-ketone **50b** is obtained. Cleavage of the product with a weak acid provides compound **51** in 88% yield. DMP will oxidize the 3-hydroxy derivative **51** to the corresponding 3-ketolide, which is a potential intermediate for the preparation of 8-fluorinated erythromycin cyclic 2´, 3´-carbamates (vide supra) [21].

Antibacterials: The MIC values against *ATTCC 25923* and *E. coli* 2592 show low activity in comparison with clarithromycin as the reference macrolide. The low potency may in part be due to loss of the basic tertiary amino center in the desosamine sugar moiety that has been converted to the *N*-acylated species **51**.

### 2.10. C9–C10 Unsaturation

**Scheme 13:**
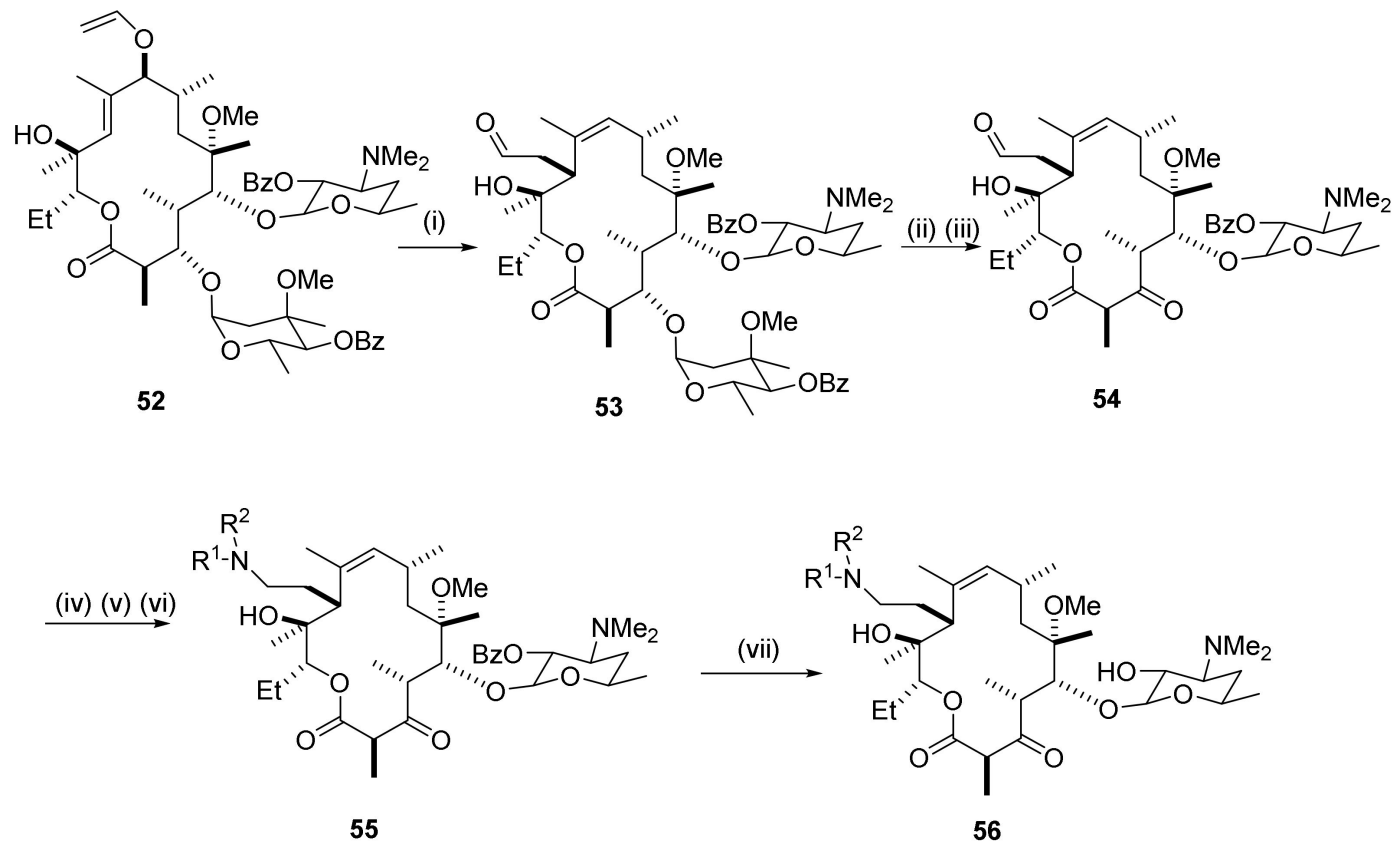
Reagents and conditions: (**i**) Toluene, heat; (**ii**) acid; (**iii**) oxidation; (**iv**) R^1^R^2^NH, NaBH_3_CN, EtOH; (**v**) acid; (**vi**) oxidation; (**vii**) MeOH.

A Claisen rearrangement of the 9-vinyl ether **52** affords C9-C10 unsaturation when compound **52** is thermolyzed in an aprotic solvent to afford the 9,10-alkene **53** (Scheme 13) [22]. Further treatment with acid at elevated temperature removes the C3-cladinose sugar. Selective oxidation of the 3-hydroxy group by DMP affords the aldehyde **54**. Methanolysis removes the protecting group at the 2′-position. Reductive amination using sodium cyanoborohydride in ethanol affords the amine **55** that is deprotected by methanolysis to furnish the 9,10-unsaturated macrolide **56**.

Antibacterials: Biodata are not presented in the report.

### 2.11. 9-Azamacrolides

**Scheme 14:**
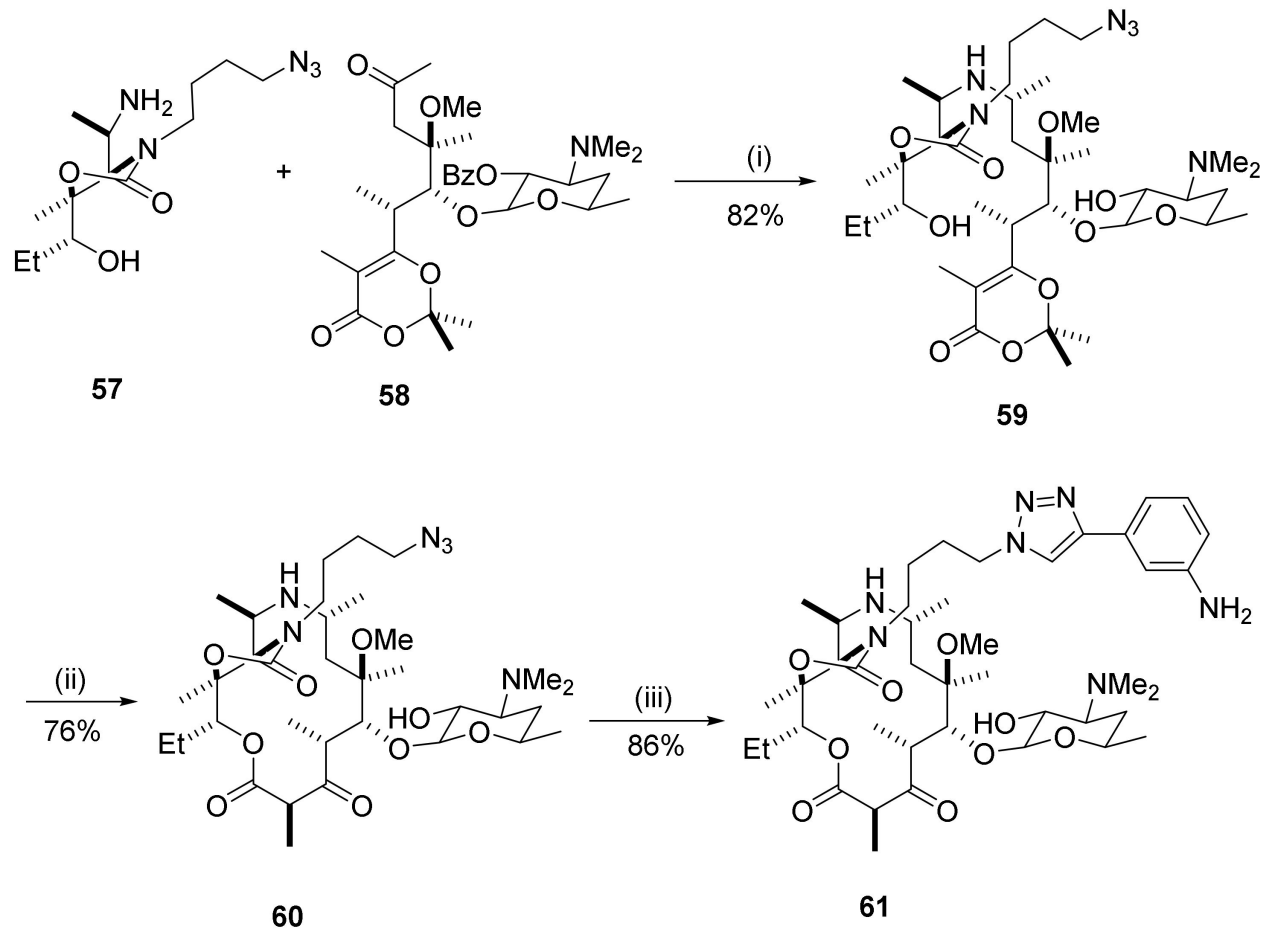
Reagents and conditions: (**i**) NaBH_3_CN; (**ii**) PhCl, 1 mM, 132 °C; (**iii**) Cu_2_SO_4_, Na-*L*-ascorbate.

Replacement of the 9-keto carbon by an amino nitrogen atom provides 9-azamacrolides. A total synthesis of the 9-azamacrolide **60** is shown in Scheme 14. The building blocks **57** and **58** are joined in a reductive amination reaction of the keto function to provide the linear chain product **59**. The latter is a substrate for macrocyclization by thermolysis (1 mM in chlorobenzene) to yield the macrolactone **60** in 78% yield [15]. The cyclization occurs without significant negative influence from the secondary amino function or the secondary alcohol. The reaction is promoted by the rigid cyclic carbamate function on the left-hand side of the molecule that brings the acyl ketene function and the reactive secondary alcohol into favorable proximity. The thermolysis proceeds through a transient acyl intermediate. Incorporation of the building block for azide-alkyne dipolar cycloaddition delivers structure **61** (which corresponds to 2-desfluorosolithromycin).

### 2.12. Transformations in the C10-Position

#### 2.12.1. Activation of the C10-Methyl Group

**Scheme 15:**
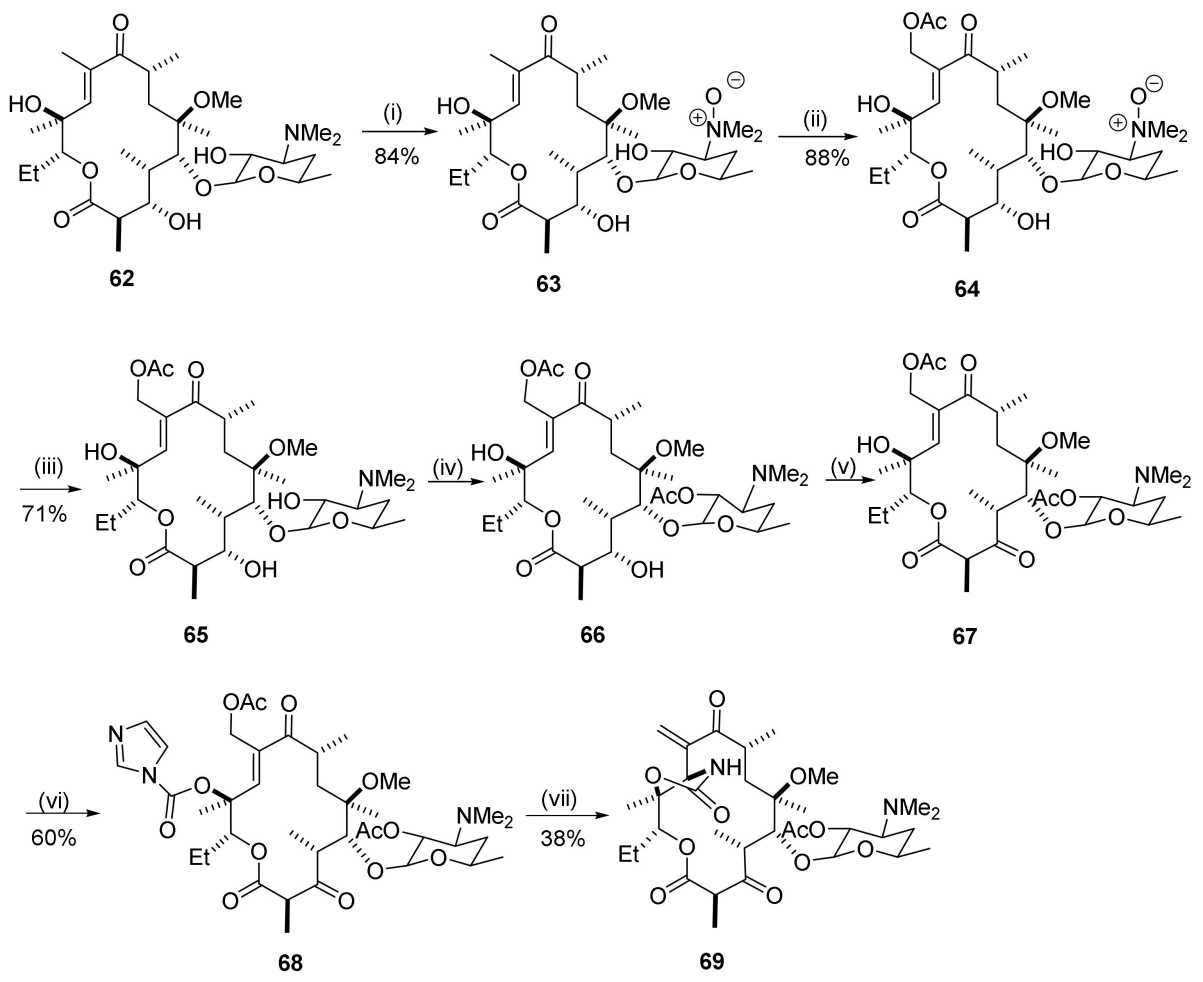
Reagents and conditions: (**i**) H_2_O_2_, MeOH, rt, 9 h; (**ii**) NCS, AcOH, rt, 20 h; (**iii**) PPh_2_PCH_2_CH_2_PPh_2_, THF, reflux, 20 h; (**iv**) Ac_2_O, NEt_3_, rt, 17 h; (**v**) DMP, CH_2_Cl_2_, rt, 3 h; (**vi**) CDI, NaH, 0 °C to rt, 17 h; (**vii**) NH_3_(aq), MeCN, THF, rt, 48 h.

**Scheme 16:**
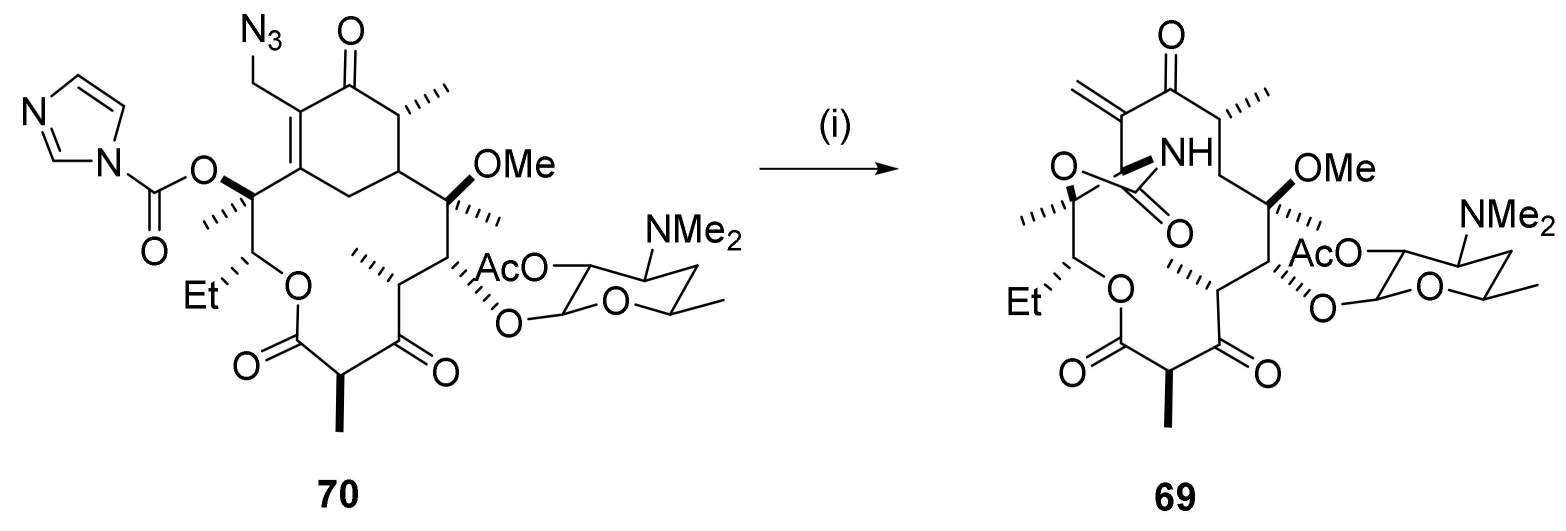
Reagent and conditions: (**i**) NH_3_(aq), MeCN, THF, rt, 17 h.

Reactions leading to transformations of the C10-methyl group in the erythromycin scaffold are illustrated in Scheme 15. The 6-methoxy function in clarithromycin may be regarded as a protected 6-hydroxy group in erythromycin. Before the reaction, clarithromycin is transformed into the 10,11-anhydro-*O*^6^-methylerythromycin substrate **62** (Scheme 15) and the tertiary 3′-amino group is oxidized chemoselectively to the *N*-oxide **63** using hydrogen peroxide in methanol [23]. The reaction in methanol proceeds almost to completion, affording the oxide in 84% yield. The *N*-oxide function serves to protect the amino-nitrogen in the ensuing reaction. Use of NBS or NCS for halogenation of the oxide **63** affords the allylic acetate **64**. NCS seems to be the better reagent (88% yield). This is a key reaction for the chemical activation of the C10-methyl group. With NBS, some brominated byproducts may be isolated. Deoxygenation of the *N*-oxide by 1,2-bis (diphenylphosphanyl) ethane affords the amine **65**. Triphenylphosphane is an alternative reagent for deoxygenation. The 2′-hydroxy sugar group is acetyl-protected (**66**) and the acetate is oxidized chemoselectively by DMP to afford the ketolide **67**. CDI in the presence of NaH in THF affords the 12-acylimidazole **68**. Further treatment with aqueous ammonia in acetonitrile leads to a two-step cyclization by the Baker procedure. The internal Michael addition results in acetate elimination to deliver the 10-methylene product **69** [23,24]. The stereochemistry in the cyclic carbamate formation is established by NMR spectroscopy and has been verified by single-crystal X-ray analysis. *N*-Substituted cyclic carbamates are formed (vide infra) with primary amines. Other allylic leaving groups at the C10-carbon will afford 10-methylene products. An example is provided by the 10-azidomethyl derivative **70** in a reaction with aqueous ammonia (Scheme 16) [23].

Antibacterials: MIC data related to reference [24] are available in reference [25].

#### 2.12.2. C10-Methyl Aminations

**Scheme 17:**
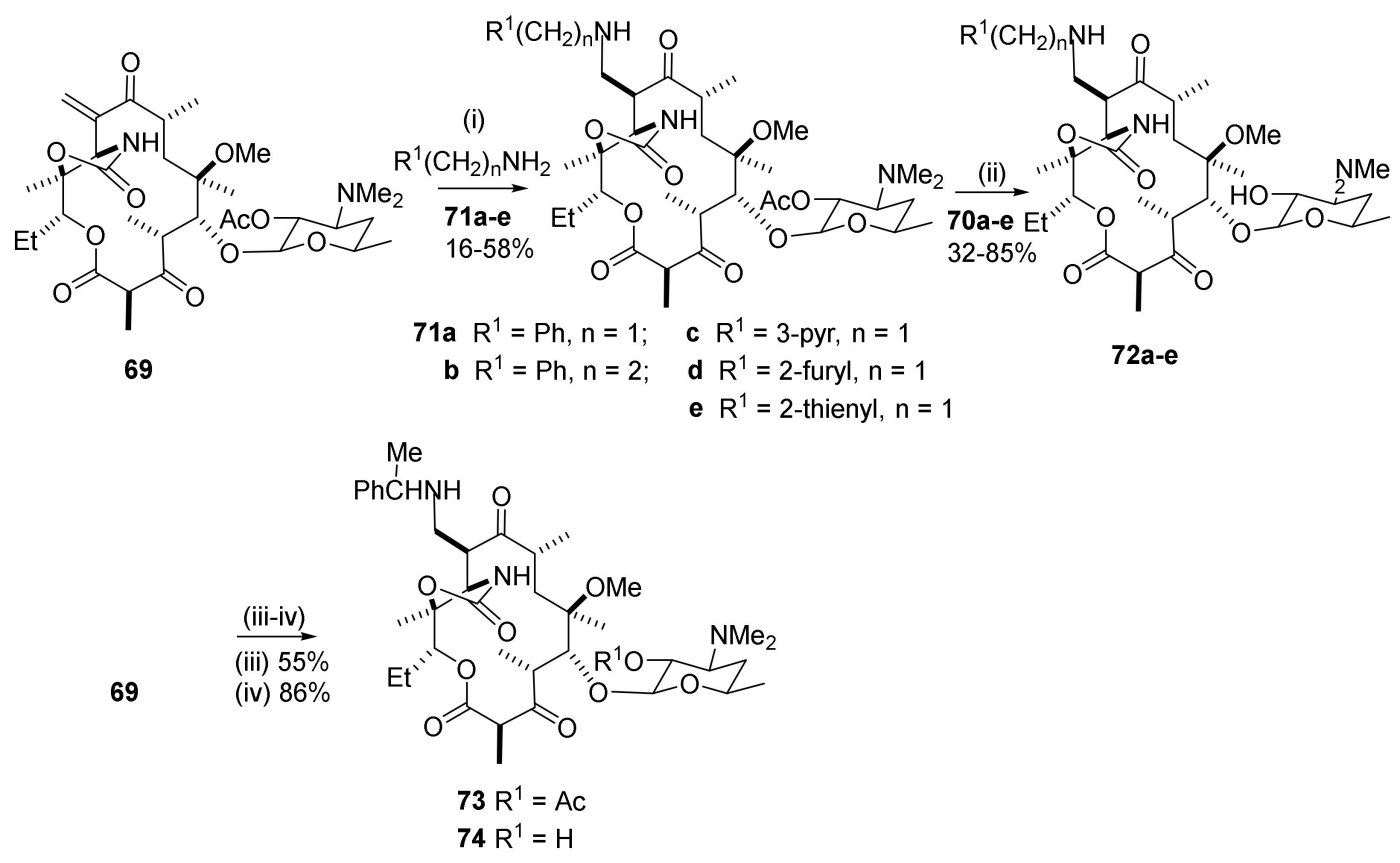
Reagents and conditions: (**i**) R^1^CH_2_NH_2_, THF, reflux, 48 h; (**ii**) MeOH, rt. 14 h; (**iii**) PhCH(Me)NH_2_, THF, reflux 60 h; (**iv**) MeOH, rt, 14 h.

The C10-methylene group in structure **69** is part of a π-electron-deficient α,β-unsaturated carbonyl system that reacts with heteroatom nucleophiles (Scheme 17) [25]. Benzylamine is a good nucleophile in the conjugated amine addition. Phenethylamine reacts in the same manner. Pyridine-3-amine has a π-deficient system with deactivation of the amino group. The yield of the adduct **71c** is low. The furyl-2- and thienyl-2-amines are π-excessive systems and afford the furyl derivative **71d** and the thienyl derivative **71e** in high yields. Cleavage of the ester group in compound **71** by methanolysis affords the 2′-hydroxy compound **72**. Only one C10-stereoismer is obtained. The product is identified by its diagnostic signal in ^1^H NMR from H-11, which resonates as a singlet at ca. 3.9 ppm (3.82, 3.91, and 3 × 3.93 ppm) and is, therefore, assigned the (*R*)-configuration. In closely related clarithromycin derivatives, it has been observed that the vicinal H-11 proton in the (10*S*)-isomer resonates as a doublet, but as a singlet in the (10*R*)-isomer. The C10-epimeric preference will depend on structural features as well as experimental conditions. The natural (10*R*)-isomer is thermodynamically more stable than its (10*S*)-isomer (vide supra). The reactivity is affected by the nucleophilicity of the amino nitrogen. Aniline fails to form adducts under the conditions used in the reactions with alkylamines. Non-bonded interactions are also important. The secondary *N*-methylbenzylamine fails to form adducts. Shifting the methyl group to the α-benzyl carbon, however, gives an active amine affording the adduct **73**.

Antibacterials: The in vitro MIC values against strains of the respiratory pathogens *S*. *pneumonia* and *S*. *aureus* show antibacterial potencies and profiles close to the values of clarithromycin together with improved activities against efflux-resistant *S*. *pneumonia* BAA1402M (mef), as well as against inducibly resistant strains of *S*. *aureus* BAA976M (mef), and some improvement towards BAA977 iMLS (erm). The MIC values show that the C10-methylene compound is less active than the reference compound, clarithromycin. Activity is greatly improved for the more lipophilic benzylated *N*-carbamoyl homologue.

#### 2.12.3. C10-α-Heteraspirane Formation

**Scheme 18:**
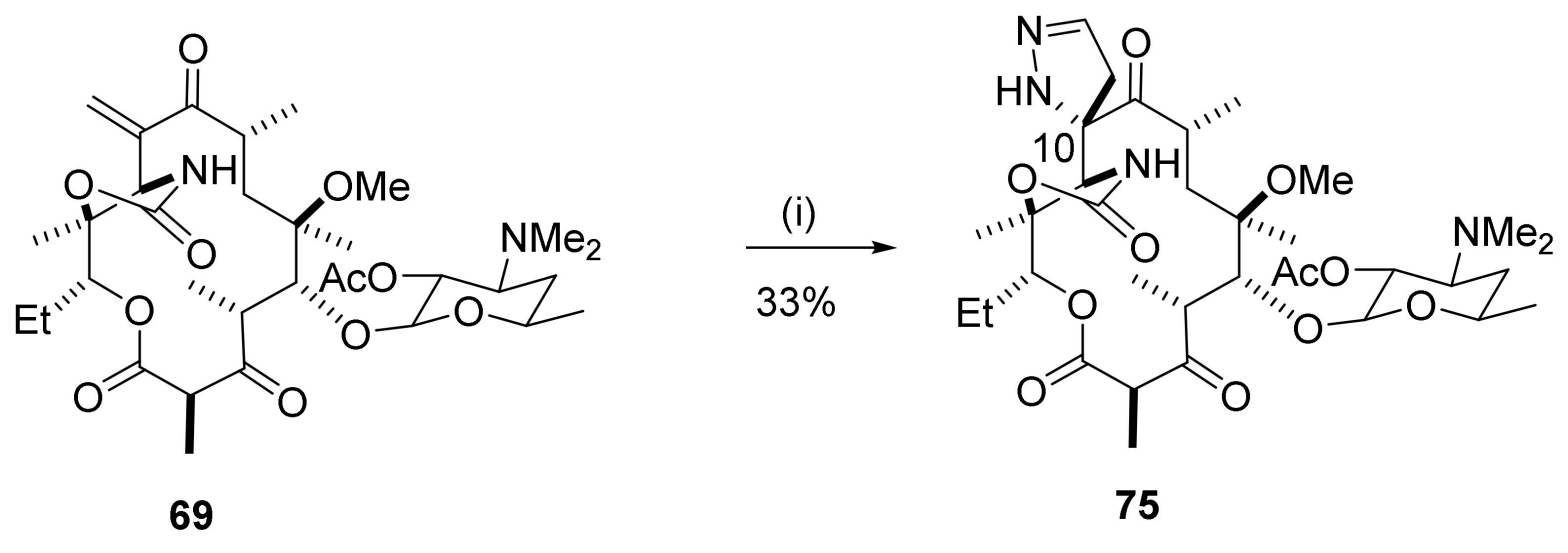
Reagents and conditions: (**i**) TMSCHN_2,_ CH_2_Cl_2_, 0 °C- rt, 72 h.

The C10-carbon is transformed into a quaternary spirocarbon by (3 + 2) dipolar cycloaddition reactions [25]. Scheme 18 shows a reaction between trimethylsilyl diazomethane and substrate **69** in dichloromethane. With electron-deficient alkenes, the nucleophilic diazoalkene adds to the more electrophilic β-carbon of the alkene. The initially formed 3*H*-4,5-dihydropyrazole is unstable and is isomerized to 1*H*-4,5-dihydropyrazole. The cycloadduct **75** is obtained in 33% yield after 48 h at 0 °C. A significant non-bonded interaction from the β-substituent of the macrolide core may, in part, explain the slow reaction. An elimination reaction in the initially formed spirane affords the final product **75**. H-11 in **75** has no vicinal protons for coupling in NMR and resonates as a singlet at 3.56 ppm. Only one singlet is seen for the H-11 proton in support of a pure epimer. Two NH signals are present as singlets at 5.50 and 6.33 ppm, in accordance with structure **75**.

Antibacterials: The MIC values for the C10-spiroketolide do not differ significantly from the figures for the reference compound, clarithromycin.

#### 2.12.4. C10-Carbylation Reactions

**Scheme 19:**
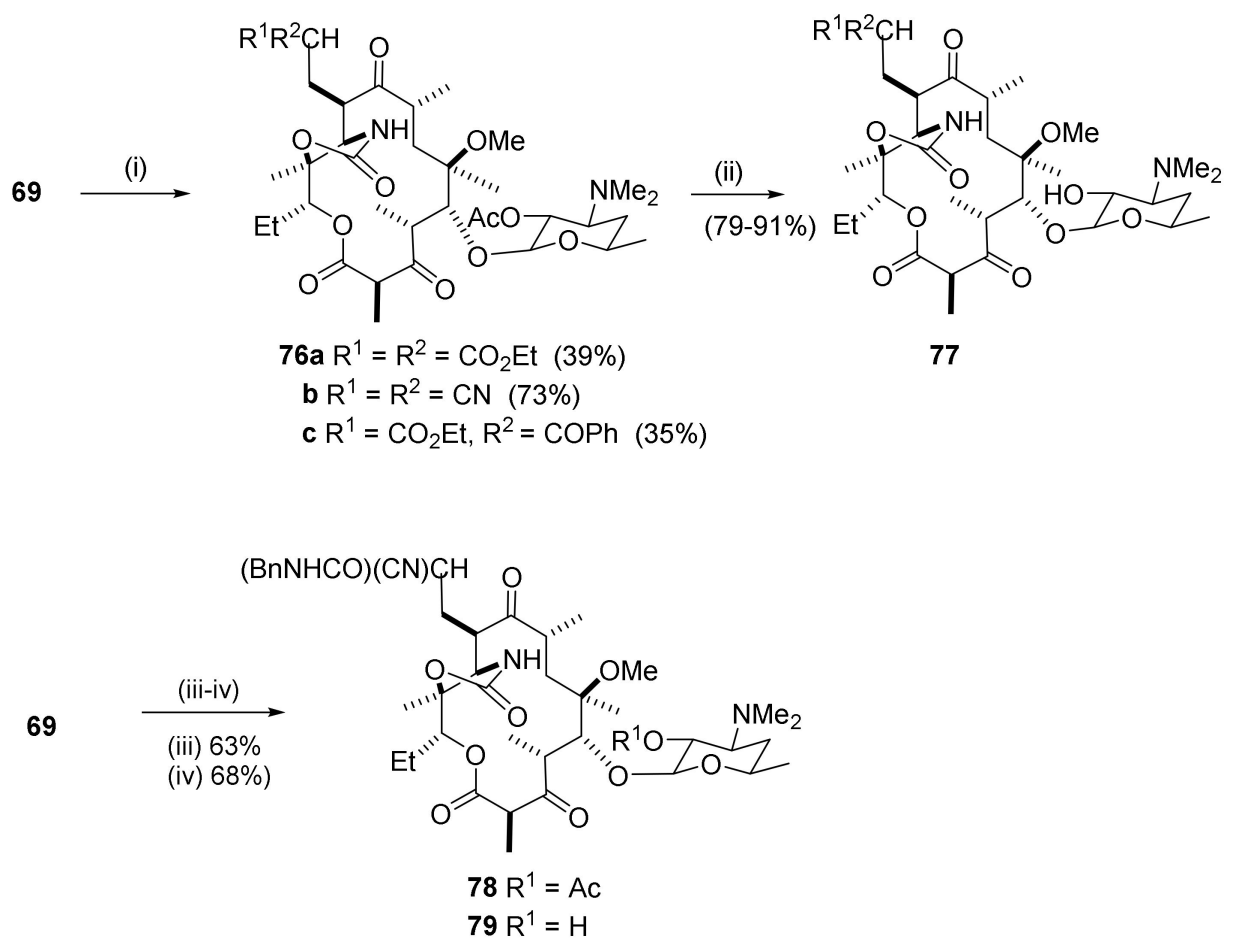
Reagents and conditions: (**i**) R^1^CH_2_R^2^, NaH, THF, 0 °C to rt; MeOH, rt, 14 h; BnNHCOCH_2_CN, NaH, THF, 0 °C to rt.; (**iii**) PhCH(Me)NH_2_, THF, reflux, 60 h; (**iv**); MeOH, rt.

Stabilized carbanions from the sodium salts of diethyl malonate, malononitrile, or ethyl 3-oxo-3-phenylpropanoate react at the C10-hinged methylene carbon to afford adducts **76** (Scheme 19). *N*-Benzyl-2-amino-2-cyanoacetamide reacts similarly with formation of the adduct **78** that is hydrolyzed to the alcohol **79** [25].

Antibacterials: The MIC values for the malonyl esters are close to the values for the reference compound, clarithromycin.

#### 2.12.5. C10-Trans-Coupling by Transition Metal Catalysis

**Scheme 20:**
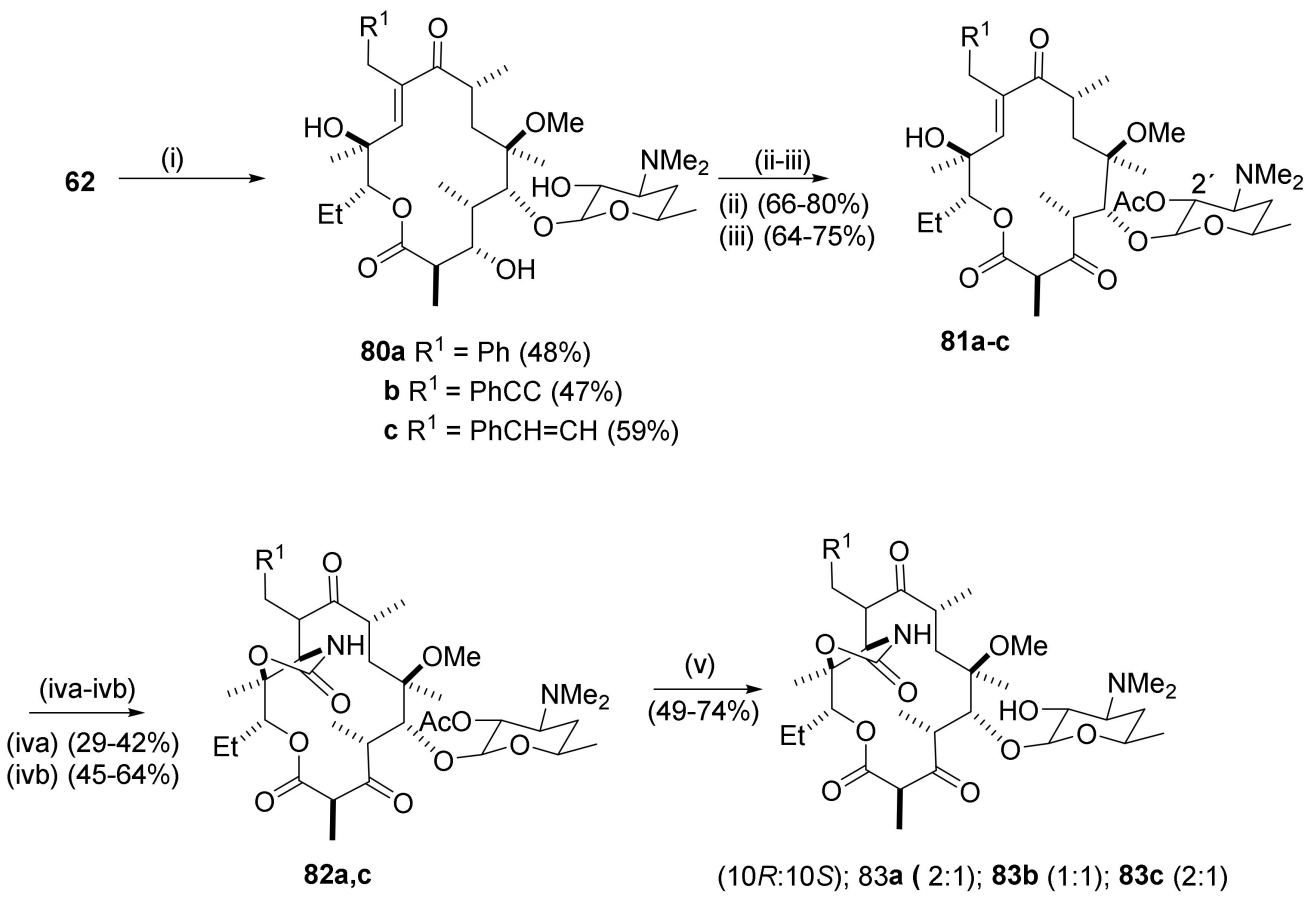
Reagents and conditions: (**i**) Bu_3_SnPh, Pd_2_dba_3_, CHCl_3_, NMP, 90 °C, 14 h; (**ii**) Ac_2_O, NEt_3_, CH_2_Cl_2_, rt, 17 h; (**iii**) DMP, CH_2_Cl_2_, rt, 3 h; (**iv**) (**a**) CDI, NaH, THF, 0 °C, 20 h, (**b**) NH_3_(aq), MeCN, THF, rt, 48 h; (**v**) MeOH, rt, 14 h.

Palladium-mediated trans-coupling reactions under Stille conditions with the acetate **62** afford aryl, alkenyl, and alkynyl products. Formation of 10-phenyl, 10-phenylethynyl, or 10-phenylethenyl products **80** is shown in Scheme 20. Trans-coupling under Suzuki conditions using organoboranes may be preferable to the Stille conditions when it is important to exclude any toxic tin-containing impurities in products that are to be used for clinical studies. The coupling product **80** is protected by acetylation of the 2′-hydroxy group before the 3-OH group is oxidized under DMP conditions to afford the 3-oxo derivative **81**. A subsequent reaction with CDI gives the corresponding 12-imidazolylcarbonyl ester that affords target compound **82** in aqueous ammonia. Methanolysis provides the deprotected product **83** as a mixture of the two C10-epimers. Separation or partial separation is achieved by chromatography. The major isomer has the (10*R*)-configuration. The isomer distribution in **83a** and **83c** is 2:1, and in **83b**, it is close to 1:1. [25].

Antibacterials: The MIC values for the phenyl derivative are similar to the values for the reference compound, clarithromycin.

**Scheme 21:**
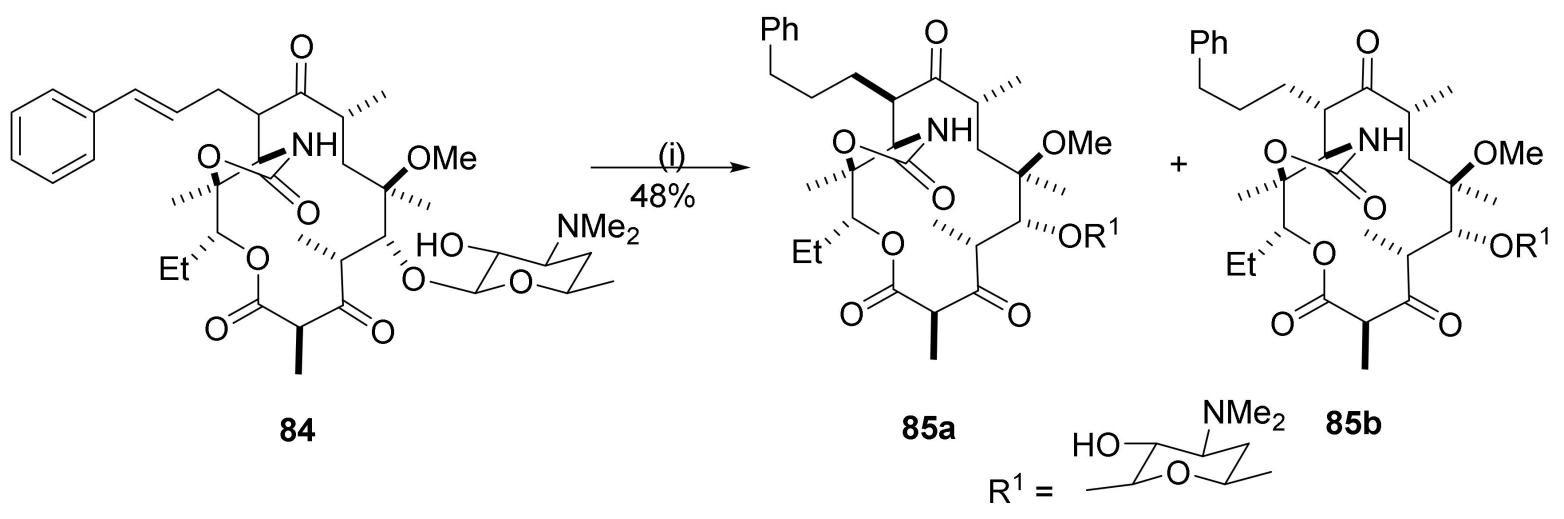
Reagents and conditions: (**i**) Pd/C, H_2_, MeOH, rt, 24 h.

Abbreviation: NMP, *N*-methylpyrrolidin-2,5-dione. Unsaturation in the bridge can be removed by catalytic hydrogenation, as in the saturation of substrate **84** [24]. The C10-epimeric mixture **84** affords the saturated C10-epimers **85a** and **85b** (Scheme 21).

**Scheme 22:**
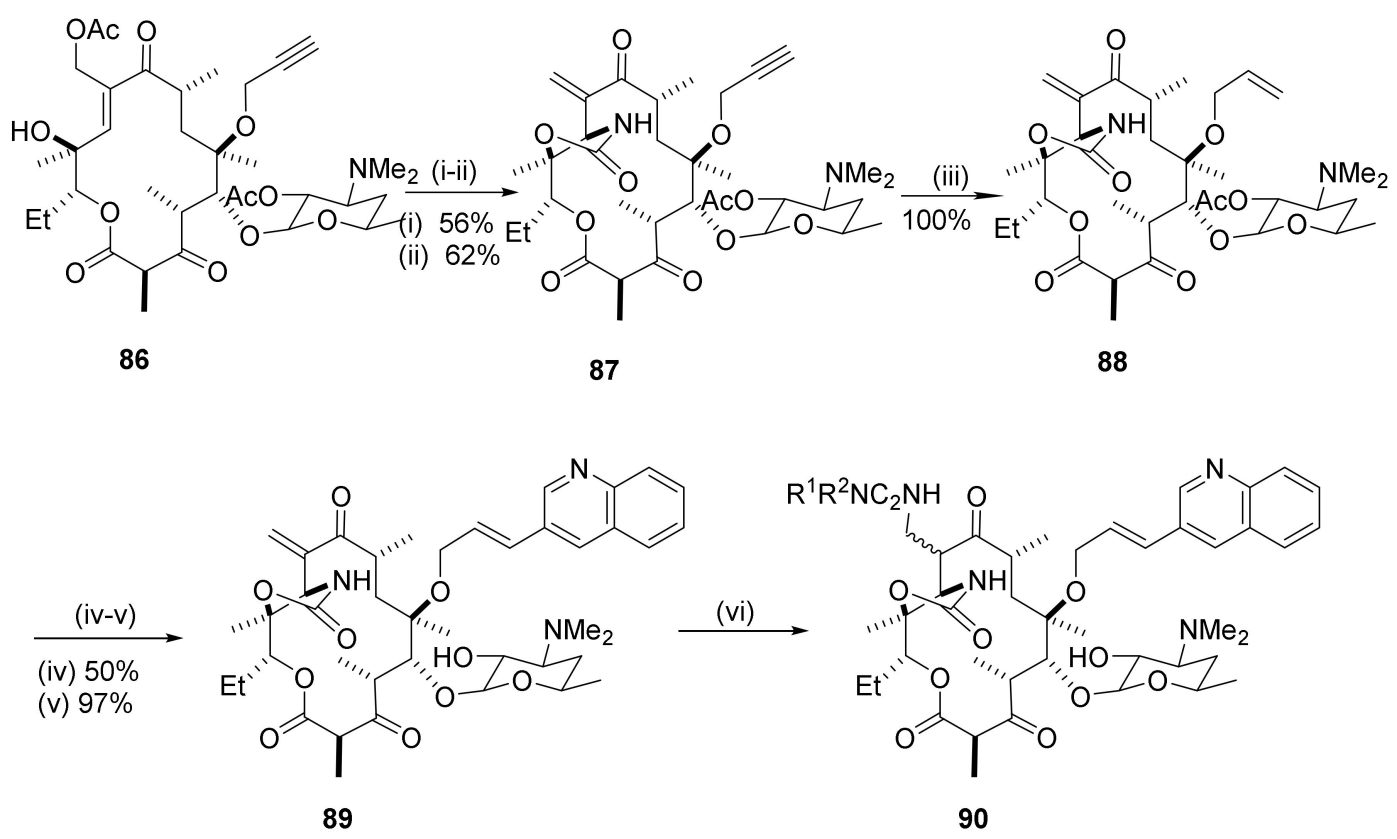
Reagents and conditions. (**i**) NaH, CDI, THF, rt, 3 h; (**ii**) NH_3_(aq), MeCN, THF; (**iii**) Lindlar cat., quinoline, EtOAc, H_2_, rt, 6 h; (**iv**) 3-Br-quinoline, MeCN, Pd(OAc)_2_, P(toluene)_3_, NEt_3_, 90 °C, 22 h; (**v**) MeOH, rt, 22 h; (**vi**) NH_2_C_2_NR^1^R^2^, MeCN, rt, −100 °C.

Structure **90** in Scheme 22 has the same ether-sidechain as cethromycin (**E,** Figure 1) compound **E**) [26]. The synthesis of substrate **86** follows largely the methodology developed for the preparation of the clarithromycin ketolide **67** outlined in Scheme 13. Acylation with CDI in the presence of sodium hydride and cyclization of the product with ammonia affords the cyclic carbamate **87**. The triple bond in the propargyl ether moiety is reduced to a double bond under Lindlar conditions. The resultant allyl intermediate **88** is subjected to trans-coupling with 3-bromoquinoline in acetonitrile in a reaction promoted by Pd(OAc) and tri-*o*-tolylphosphine to produce the 6-(quinolin-3-yl) Heck product **89**. Adduct formation between diamine reactants and **89** affords the C10-diamine **90** by analogy to the procedure in Scheme 17.

Antibacterials: The in vitro MIC values show little or no alteration in potency compared to the values for cethromycin against the strains *S. pneumonia* ATCC49619, *S. pneumonia* ATCC1402, and *S. pneumonia* ATCC1407.

#### 2.12.6. Reactions in the C12-Position

**Scheme 23:**
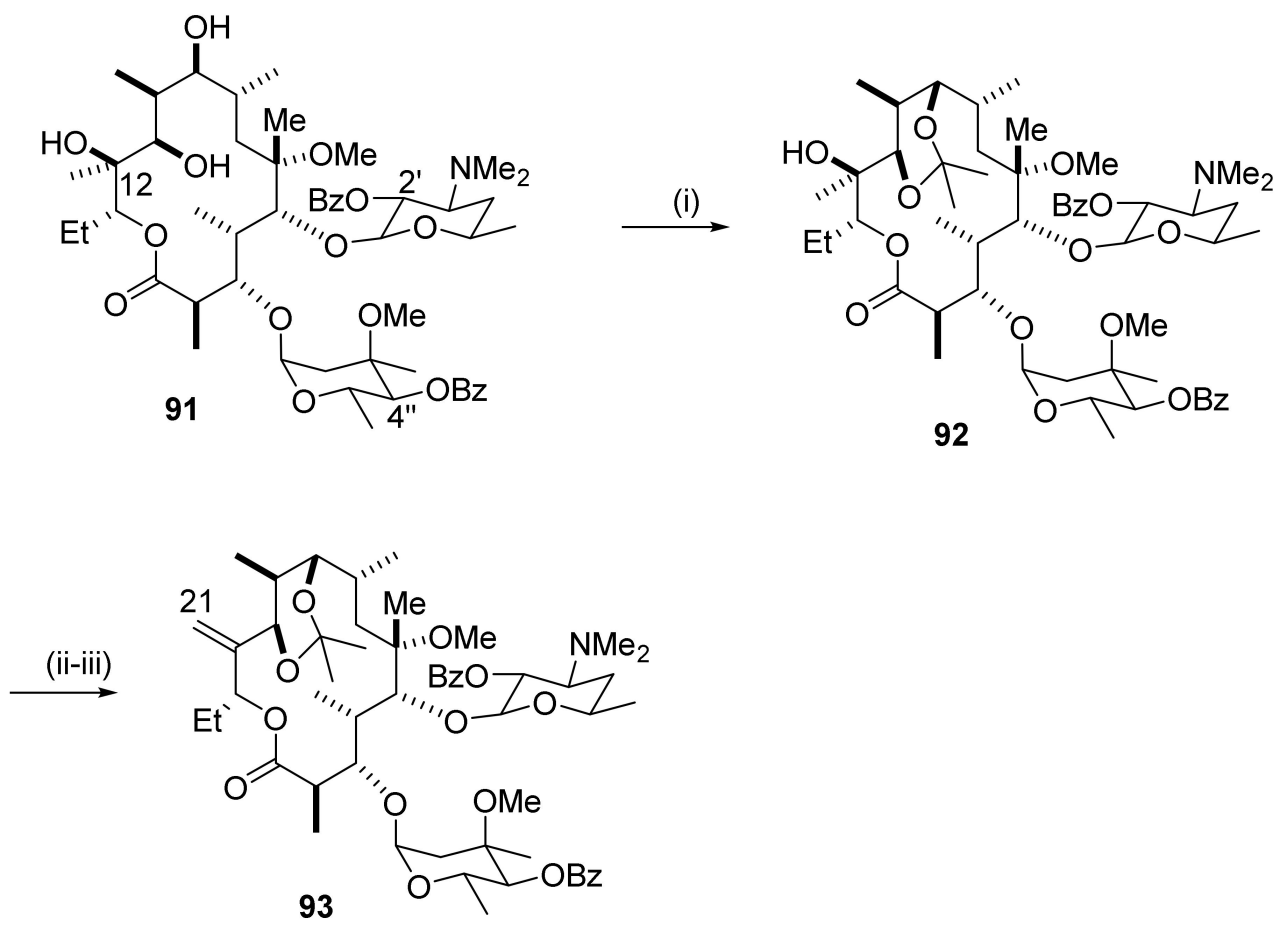
Reagents and conditions: (**i**) Me_2_C(OMe)_2_,TsOH-pyridine, acetone, reflux; (**ii**) SOCl_2_, NEt_3_, EtOAc; (**iii**) TsOH, MeCN, H_2_O, 68 °C.

Scheme 23 shows synthesis of a C12-methylene ketolide **93** [27]. For the synthesis, the 2′,4″-dihydroxy groups in clarithromycin are protected by benzoylation and the 9-keto group is reduced by sodium borohydride to afford the 9-hydroxy intermediate **91**. The latter is protected as a cyclic ketal **92** using TsOH as the catalyst under reflux in acetone. The ketal in ethyl acetate is reacted with sulfonyl chloride and triethylamine and subsequently, with TsOH in MeCN at 60 °C to afford the C12-methylene ketal **93**. The C12–C21 double bond provides a handle for introduction of novel groups into the C12-position in the macrolide core.

**Scheme 24:**
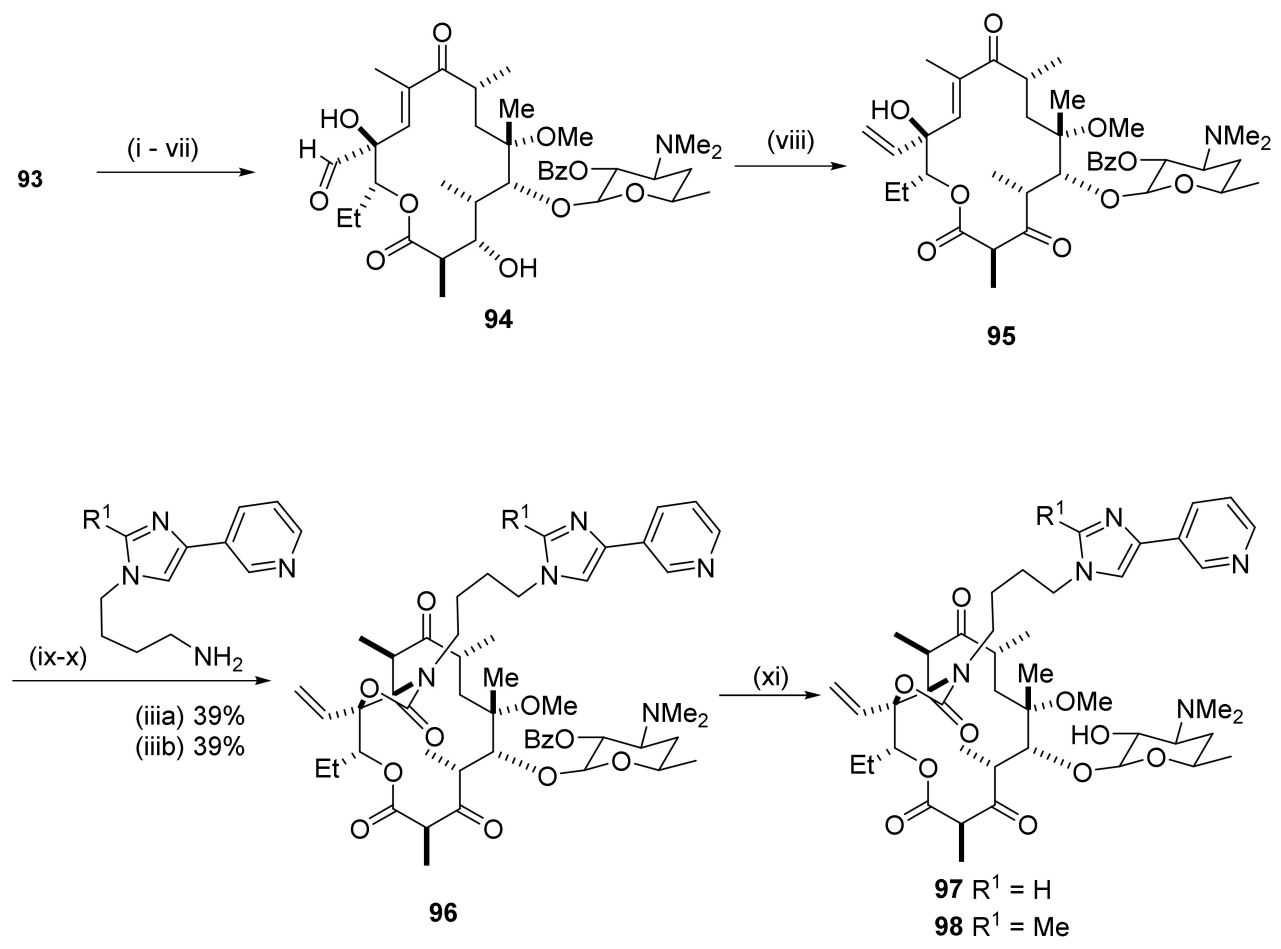
Reagents and conditions: (**i**) TsOH, MeCN, H_2_O, 65 °C; (**ii**) OsO_4_, NMO, acetone, H_2_O; (**iii**) AcCl, DMAP, pyridine, (**iv**) DMP, CH_2_Cl_2_, −10 °C; (**v**) MsCl, pyridine; (**vi**) DBU, acetone, rt, −65 °C, 16 h; (**vii**) Me_2_S, NCS, THF, −13 °C (**viii**) MeP(Ph)_3_Br, KN(TMS)_2_, THF, toluene, −60 °C to rt; (**ix**) CDI, THF, NaH, 0 °C; (**x**) Het-amine, MeCN, H_2_O. 14 h; (**xi**) MeOH, 70 °C. Abbreviations: NMO, *N*-methylmorpholine oxide; DBU, 8-diazabicyclo[4,5.0]undec-7-ene.

Introduction of a vinyl group into the 12-position is shown in Scheme 24. A number of reaction steps from the 12-methylene compound **93** (Scheme 23) affords the 12-formyl-12-hydroxy intermediate **94** [27]. A Wittig reaction at the 12-formyl group using (methyl) triphenyl phosphonium bromide together with potassium bis (trimethylsilyl) amide as base affords the vinyl derivative **95**. Further reactions using the Baker protocol afford the C12-vinyl, C11-C12 carbamate ketolide **96**. Methanolysis provides the deprotected 12-vinyl compound **97** and its 2-methyl homologue **98**. The former is a 12-vinyl homologue of telithromycin. Several analogues are available where the sidechain is substituted in either the 5-membered or the 6-membered rings.

Antibacterials: The C12-methylene ketolides possess potent antibacterial activity against a range of pathogens. The spectrum includes activity against *S. pneumonia* strains with the mef and erm genes. The heterocyclic moieties are tethered to the cyclocarbamate nitrogen atom via a four-carbon spacer affording compounds with potencies similar to that of telithromycin against *S. aureus* and susceptible *S. pneumonia*.

**Scheme 25:**
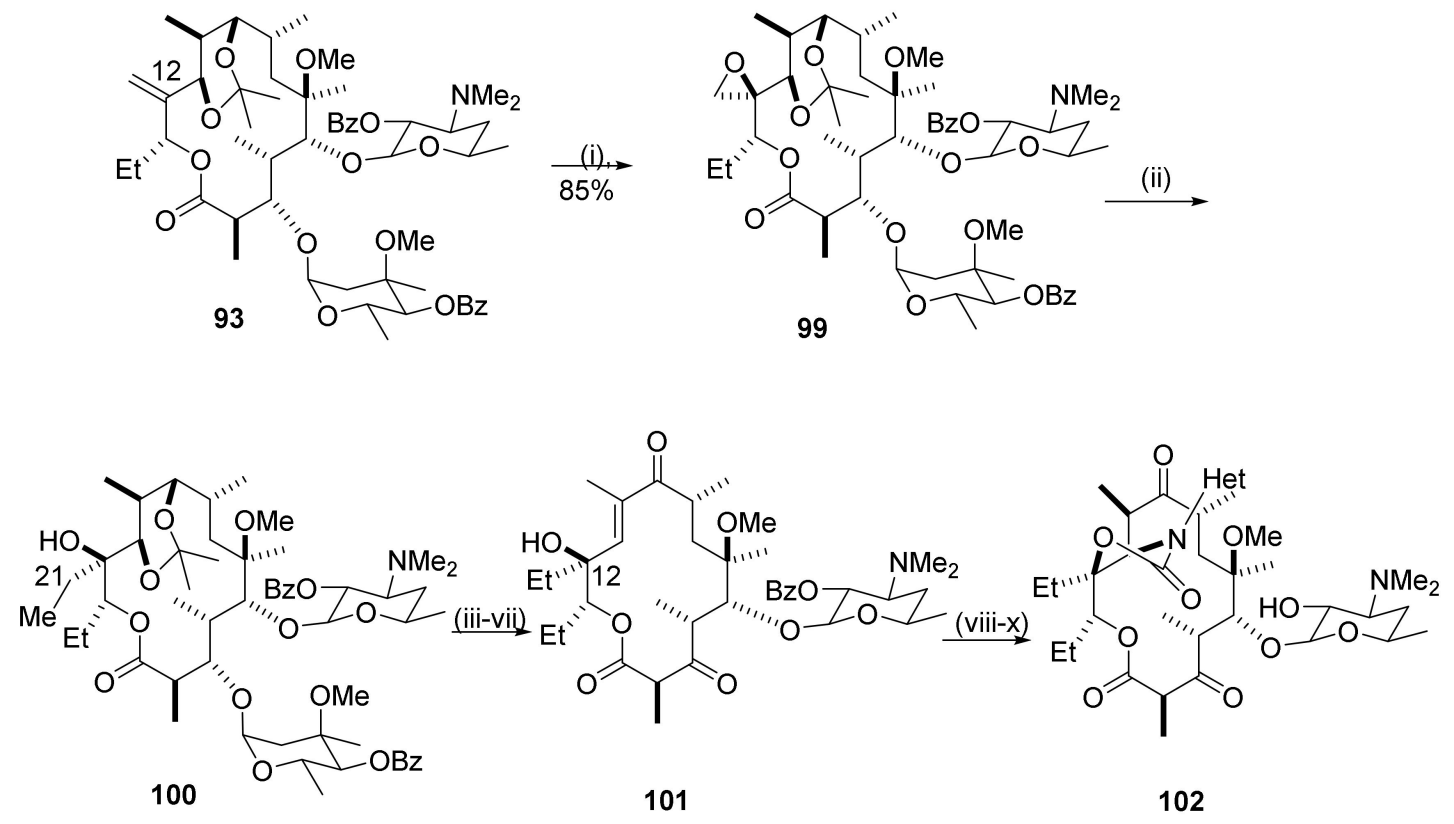
Reagents and conditions: (**i**) (a) MCPBA, CH_2_Cl_2_, rt, 21 h, (b) 2 M NaH/SO_3_(aq), rt, 30 min; (**ii**) (a) Me_2_S.CuBr, −78 °C, (b) MeLi, −78 °C, (c) epoxide, 0–5 °C, 12 h; (**iii**) TsOH/pyridine, MeCN:H_2_O (2:1), 78 °C 48 h; (**iv**) DMP, CH_2_Cl_2_, −5 to −10 °C, 30 h; (**v**) (a) MsCl, pyridine, 0 °C—rt, 18 h, (b) DMU, acetone, rt, −40 °C, 40 h; (**vi**) 3 M HCl, MeCN. rt, 22 h: (**vii**) DMP, CH_2_Cl_2_, rt, 2 h; (**viii**) NaH, CDI, THF, −15 to 0 °C, 25 min; (**ix**) Het-amine, 78 °C, 40 h; (**x**) MeOH, 70 °C, 22 h. Abbreviation: MCPBA, *m*-chloroperbenzoic acid.

A formal replacement of the natural C12-methyl group in the erythromycin core with an ethyl group is illustrated in Scheme 25 [28]. The structural modifications start with epoxidation of the C12-methylene macrolide **93** using MCPBA [29]. Oxidation of the tertiary amino function occurs concurrently. The *N*-oxide is reduced by sodium thiosulfate in aqueous THF to afford epoxide **99**. The epoxidation is stereoselective and occurs from the *beta* face of the macrocycle. Opening of the epoxide ring with dimethylcuprate adds a methyl group at C21 to afford the 12-ethyl derivative **100**. Further transformations, as indicated in Scheme 25, afford the unsaturated intermediate **101** that is subjected to the Baker protocol for conversion to the 12-ethyl target compound **102**.

Antibacterials: C12-ethyl ketolides with tethered heteroaromatic fused bicycles have potencies similar to telithromycin against *S. aureus*, *S. pyogenes*, the susceptible *S. pneumonia* mef gene containing *S. pneumonia* strains, and *H. influenzae*. They have a weaker potency than the reference compound, telithromycin, against *S. epidermidis*, *E. faecalis*, and the erm gene containing *S. pneumonia* strain A in an in vivo mouse infection model.

#### 2.12.7. Reactions in the C13-Position

**Scheme 26:**
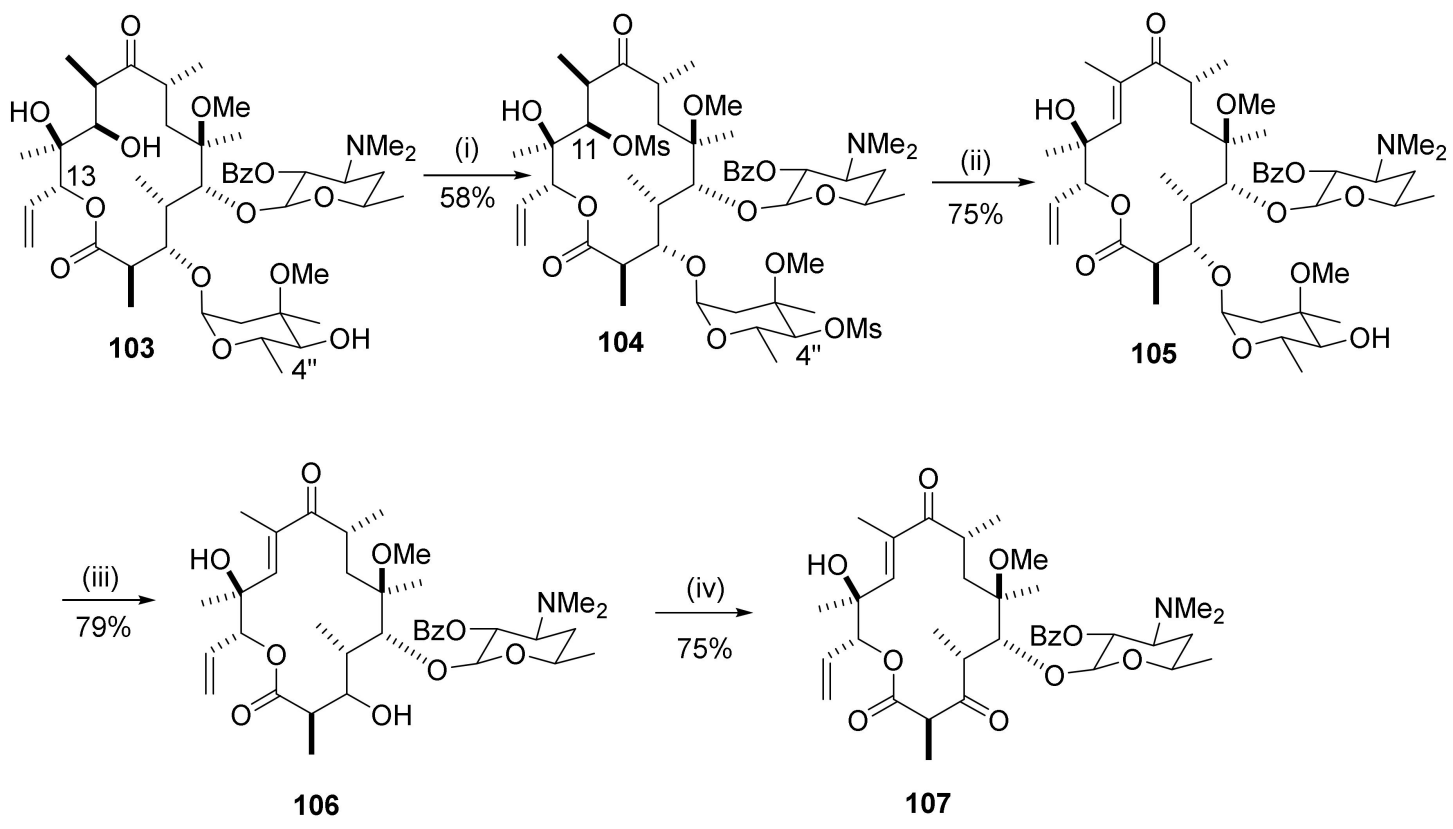
Reagents and conditions: (**i**) MeSO_2_Cl, pyridine, rt, 24 h; (**ii**) DBU, acetone, rt, 24 h; (**iii**) 3 M HCl, MeCN, rt, 22 h; (**iv**) DMP, CH_2_Cl_2_, rt, 1 h.

The C13-ethyl group is chemically inactive in the naturally occurring members of the erythromycins. A C13-vinyl analogue, however, provides a versatile intermediate for incorporation of several novel groups at the C13-position in the macrolide (Scheme 26). Fortunately, 13-vinyl compound **103** can be prepared indirectly by fermentation in a precursor-directed biosynthesis [29]. This methodology allows for preparation of 13-substituted derivatives that include the parent 13-ethyl compound. The 2′-*O*-benzoyl macrolide **103** is a substrate in the preparation of the 14,15-dehydroerythromycin product **107** (Scheme 26). Mesylation provides the 11-*O*-mesylate **104** that is also mesylated at the 4″-hydroxy group. The 11-*O*-mesyl group is eliminated by treatment with a base such as DBU to afford the 10,11-alkene **105**. Hydrolysis under acidic conditions removes the 3-glycosyl function to afford the 3-hydroxy derivative **106**. A subsequent DMP oxidation of the 3-OH group provides the 14,15-dehydroerythromycin A ketolide **107**.

Antibacterials: MIC data not accessible.

#### 2.12.8. Reactions in the C15-Position

**Scheme 27:**
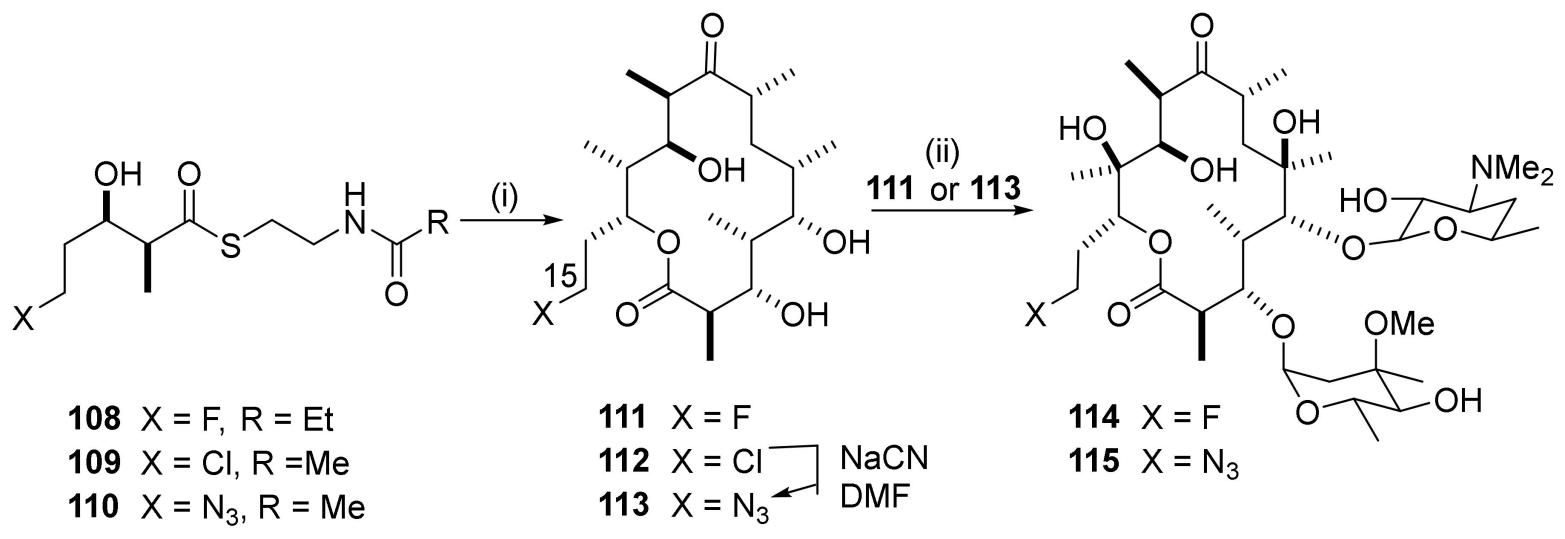
Reagents and conditions: (**i**) *Strept. coelicolor* CH999/pJRJ2; (**ii**) *Sac. Erythraea* K39-14V.

Chemobiosynthesis has been used to prepare analogues of erythromycins with functional groups in the 15-position. The reactions are illustrated by the preparations of 15-fluoro, 15-chloro, and 15-azido derivatives (Scheme 27) [30]. The preparation starts with synthesis of the racemic diketide thioesters **108–110** that are converted into the corresponding 6-deoxyerythronolide B analogues **111–113** using *streptomyces coelicolor* CH999/pJRJ2. Conversion of the chloride **112** into azide **113** is effected by a reaction with NaN_3_ in DMF. An engineered mutant strain *Saccharopolyspora erythraea* K39-14V is used for the subsequent bioconversion of **111** and **113** into the erythromycin A glycosides **114** and **115**. Inaccessible analogues of erythromycin by traditional chemical methods are available by chemobiosynthetic techniques.

Antibacterials: The in vitro MIC values are comparable to corresponding data for erythromycin A. The data show that introduction of a chemical handle at C15 is not deleterious to antibacterial activity

#### 2.12.9. 15-Amino Macrolides

**Scheme 28:**
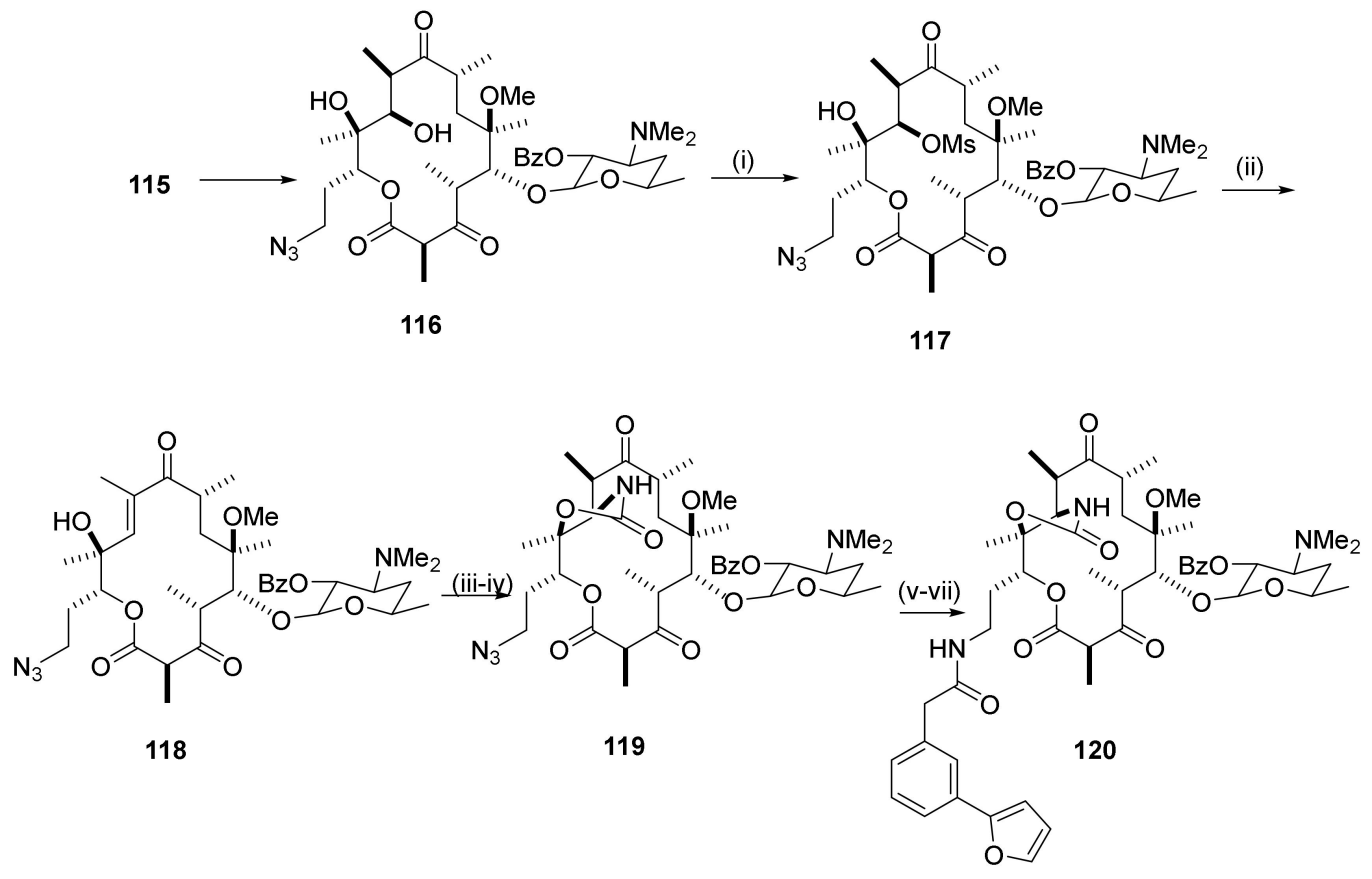
Reagents and conditions: (**i**) MsCl, pyridine, rt, 16 h; (**ii**) DBU, acetone, rt, 8 h; (**iii**) NaH, CDI, THF, −15 °C, 15 min; (**iv**) MeCN, THF, NH_3_(aq), rt, 16 h; (**v**) PMe_3_, THF, rt, 45 min; (**vi**) RCO_2_H, Me_2_N(CH_2_)_3_N = C = NEt; (**vii**) NEt_3_, MeOH, 50 °C, 14 h.

Preparation of 15-pharmacophoric amides is illustrated in Scheme 28. Selective reduction of the azido group in C15-azides affords corresponding primary amines. The 15-azido clarithromycin-derived macrolide **115** is 2′-*O*-protected by benzoylation and mesylated at the 11-hydroxy group. Treatment of the mesylate with DBU results in the elimination and formation of the 10,11-alkene **118**, which is an appropriate unsaturated intermediate for annulation by the Baker protocol to afford the 15-azido ketolide **119** [31]. The azide function is reduced selectively to amine by trimethylphosphine, and the amine is acylated using a carbodiimide coupling procedure for amide bond formation to afford pharmacophoric amides such as illustrated by structure **120**.

Antibacterials: The compounds possess antibacterial activity against Gram-positive, Gram-negative, and anaerobic bacteria, with special reference to *S. aureus*, *S. epidermidis*, *S. pneumonia*, *S. pyogenes*, and *H. influence.*

**Scheme 29:**
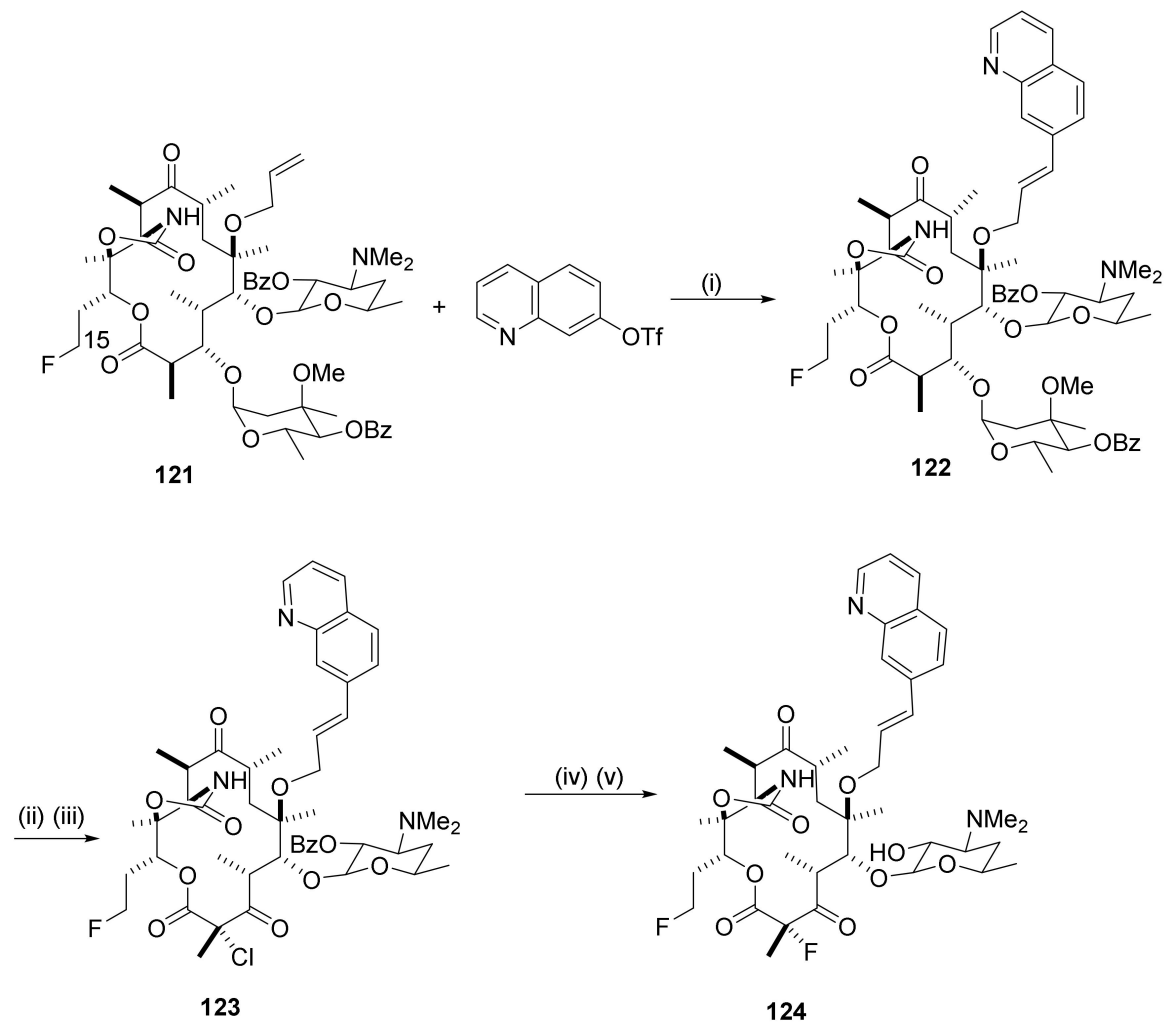
Reagents and conditions: (**i**) PdBr_2_, dppf, DiPEA, DMF; (**ii**) Et_3_SiH, TFA, CH_2_Cl_2_; (**iii**) NCS, Me_2_S, NEt_3_; (**iv**) NaHMDS, (PhSO_2_)_2_NF; (**v**) MeOH, rt. Abbreviations: DiPEA, *N*,*N*-diisopropylethylamine; dppf, bis(diphenylphosphino)ferrocene; NCS, *N*-chlorosuccinimide.

Scheme 29 illustrates synthesis of a 2,15-difluoro derivative **124**. The substrate **121** has a chemically stable C15-fluoro substituent and a chemically reactive 6-allyloxy function. The 6-allylic ether function reacts in a Pd-promoted Heck coupling with the triflate of 7-hydroxyquinoline. The C15-fluoro product **122** is enolized and converted into a silyl ether at C2 using triethylsilane. The silyl ether is chlorinated at the C2-position using NCS and is subsequently fluorinated. The product after solvolysis affords the 2,15-difluoride **124** [32].

Antibacterials: The antibacterial tests of the compounds include a rat lung pneumonia lower respiratory tract infection model. In vitro hepatotoxicity of the compounds is reported.
